# Respiratory Viruses and Virus-like Particle Vaccine Development: How Far Have We Advanced?

**DOI:** 10.3390/v15020392

**Published:** 2023-01-30

**Authors:** Ki-Back Chu, Fu-Shi Quan

**Affiliations:** 1 Medical Research Center for Bioreaction to Reactive Oxygen Species and Biomedical Science Institute, Core Research Institute (CRI), Kyung Hee University, Seoul 02447, Republic of Korea; 2 Department of Medical Zoology, School of Medicine, Kyung Hee University, Seoul 02447, Republic of Korea

**Keywords:** virus-like particle, influenza virus, respiratory syncytial virus, SARS-CoV-2, vaccine

## Abstract

With technological advancements enabling globalization, the intercontinental transmission of pathogens has become much easier. Respiratory viruses are one such group of pathogens that require constant monitoring since their outbreak leads to massive public health crises, as exemplified by the influenza virus, respiratory syncytial virus (RSV), and the recent coronavirus disease 2019 (COVID-19) outbreak caused by the SARS-CoV-2. To prevent the transmission of these highly contagious viruses, developing prophylactic tools, such as vaccines, is of considerable interest to the scientific community. Virus-like particles (VLPs) are highly sought after as vaccine platforms for their safety and immunogenicity profiles. Although several VLP-based vaccines against hepatitis B and human papillomavirus have been approved for clinical use by the United States Food and Drug Administration, VLP vaccines against the three aforementioned respiratory viruses are lacking. Here, we summarize the most recent progress in pre-clinical and clinical VLP vaccine development. We also outline various strategies that contributed to improving the efficacy of vaccines against each virus and briefly discuss the stability aspect of VLPs that makes it a highly desired vaccine platform.

## 1. Introduction

Respiratory viruses are one of the leading contributors to global mortality. Since the beginning of the 21st century, there have been several respiratory virus outbreaks in the past two decades that were accompanied by disastrous consequences, as evidenced by the recent severe acute respiratory syndrome coronavirus 2 (SARS-CoV-2) pandemic. Others, such as the respiratory syncytial virus (RSV) and influenza virus, continue to infect the lower respiratory tracts of individuals, accounting for a significant proportion of global deaths occurring in infants and the elderly [1]. These respiratory viruses can be easily transmitted from person to person via direct contact or through fomites, but they can also be transferred via air in the form of aerosols or droplets to infect susceptible hosts [2]. Numerous factors inadvertently made pathogen transmission much easier, which include technological advances and demographic and anthropogenic changes [3]. Given these circumstances, controlling the transmission of these respiratory viruses has become a necessity.

Vaccination, one of the greatest medical advancements for human civilization, is widely perceived as the most effective tool for preventing viral infectious diseases. Evidently, with the introduction of vaccines and compulsory vaccination of the general public in 18th and 19th century Europe, there was a drastic decline in smallpox-associated mortality rates [4]. Advances in molecular biology and biotechnology in the 20th century brought further improvements to the field of vaccinology, eventually paving the way to developing highly immunogenic virus-like particle (VLP) vaccines. Historically, the term “virus-like particle” was possibly first mentioned in the 1930s by Burnet, who at the time worked with bacteriophage-neutralizing antisera [5]. However, these VLPs described in the earlier literature were not necessarily the replication-deficient self-assembling viral particles that are frequently used as vaccine platforms today [6]. Currently, VLP vaccines against the hepatitis B virus and human papillomavirus have been approved by the United States Food and Drug Administration (FDA) for clinical purposes [7]. Nevertheless, VLP vaccines against respiratory viruses continue to remain under development. Furthermore, despite the breakthroughs in biomedical science, a vast majority of these efficacious vaccines remain unavailable for low-income countries, thus resulting in a staggeringly high mortality rate and disease burden [3]. Here, we review the recent progress in pre-clinical and clinical VLP vaccine development against three respiratory viruses that continue to impact the world: influenza, RSV, and SARS-CoV-2.

## 2. Influenza Virus

### 2.1. Brief History of Influenza Virus and Outbreaks

Influenza is a virus belonging to the family Orthomyxoviridae. It is a negative-sense RNA virus subdivided into four different genera: A, B, C, and D. Among these, A and B influenza viruses are of concern as they are capable of causing mortality in humans. These viruses are also capable of infecting other animals, including but not limited to birds, pigs, various vertebrates, and even aquatic animals [8]. On the contrary, influenza virus types C and D are of less concern as these are either rarely symptomatic in humans or have not been reported to infect humans at all. Various external surface proteins expressed by the influenza virus have been tested as vaccine candidates. The most studied vaccine antigens are those that are expressed on the outer membrane, such as the M2 ion channel protein, as well as the two major glycoproteins, hemagglutinin (HA) and neuraminidase (NA). To date, 18 different HA and 11 different NA subtypes have been characterized [9]. This is the result of antigenic drift occurring in these HA and NA glycoproteins, which contributed to resistance and lessened vaccine efficacy.

It is presumed that influenza outbreaks have been prevalent in human society since ancient times. While older literary sources are not as precise as current ones, the information they provided was extremely useful and suggested that influenza virus outbreaks may have occurred more than 2000 years ago. For example, the writings of Hippocrates in 412 BCE describing the “Cough of Perinthus” suggest that this may have been an influenza-like outbreak [10,11]. When reflecting on the past century, a total of four influenza-related pandemics have occurred: the 1918 A/H1N1 pandemic, which is misnomered as the “Spanish Flu,” the 1957 A/H2N2 reassortant human and avian virus that emerged in China, the 1968 A/H3N2 Hong Kong influenza, and the new A/(H1N1)pdm09 variant that first appeared in North America in the April of 2009 [12]. In each of these four pandemics, at least 10,000 lives were lost, and developing universal influenza vaccines has become a necessity. Here, we primarily focus our attention on some of the most recent VLP studies targeting human and avian influenza viruses.

### 2.2. Pre-Clinical VLP Vaccine-Induced Protection

The development of replication-deficient VLP vaccines against the influenza virus began in the 1990s. One of the earliest influenza VLP constructs was reported to be chimeric. Specifically, Layton et al. [13] demonstrated that immunizing mice with yeast-derived VLPs expressing the influenza nucleoprotein peptides could be used to induce antigen-specific cytotoxic T lymphocyte responses. However, the protective efficacy of these VLP vaccines was not investigated in earlier studies. The first chimeric VLP vaccine study to evaluate the protective efficacy of these immunogenic nanoparticles in animal models was reported by Neirynck et al. [14]. Here, the authors fused the 23 amino acid M2 extracellular peptide (M2e) of the influenza A virus to the hepatitis B virus core protein (HBc) to generate the VLPs. Mice that were immunized with these VLPs via either intranasal or intraperitoneal routes were protected in a dose-dependent manner upon subsequent challenge infection. Some studies investigated the protective efficacy of VLPs that more closely resembled the native influenza virus. Interesting results were reported from mammalian cell-derived influenza VLPs solely lacking the non-structural influenza virus proteins. Immunizing mice with the VLPs lacking both NS1 and NS2 resulted in survival rates below 10%, whereas VLPs lacking only the NS2 gene resulted in 94% survival post-challenge infection [15]. Since then, studies investigating the formation of VLP constructs using various expression systems have been conducted, and massive improvements to influenza VLP vaccine assembly have ensued. It was later revealed by Latham and Galarza [16] that the expression of the M1 protein alone was sufficient for the efficient release of viral particles in *Spodoptera frugiperda* (Sf9) insect cells. This period was followed by a plethora of studies assessing the efficacy of VLPs expressing HA, NA, or other influenza antigens on the surface of M1 proteins.

The M2e antigen has been of considerable interest in influenza vaccine development. Fusing multiple copies of the M2 ectodomain has been studied widely, using several virus backbones. Genetically fusing copies of the M2e antigen to the C terminal end of the *Macrobrachium rosenbergii* nodavirus capsid protein was sufficient to confer protection in BALB/c mice. This chimeric vaccine, without the use of adjuvants, conferred protection against both H3N2 and H1N1 challenge infection [17]. Similar results were obtained from bacteriophage-based VLP vaccines evaluated in animals, as demonstrated by AP205 bacteriophage VLPs expressing the triplets of 24 amino acid sequences of the M2e derived from H1N1, H5N1, and H11N9. This vaccine conferred protection against both homologous and heterologous virus challenge infection in mice. When the long alpha-helices of the trimeric H1 stalk proteins were coupled to these VLPs, protection against lethal homologous challenge infection was enhanced [18]. M2e-expressing VLPs based on the T4 bacteriophage was also a feasible approach. These VLP vaccines induced strong cellular and humoral immune responses without adjuvant incorporation and conferred full protection against lethal challenge infection by the A/PR/8/1934 virus [19]. In another VLP construct, the globular head domain of influenza HA from A/Puerto Rico/8/1934 (H1N1) was conjugated to P22 bacteriophage via SpyTag/SpyCatcher technology, and their immunization in mice conferred full protection against homologous challenge infection [20].

Much emphasis has been placed on HA antigens, while NA antigens, despite their importance and contribution to protection, continue to be overlooked in influenza vaccine development. Though the number of studies investigating the efficacy of NA VLPs may be lacking, some of these findings were critical to understanding their role as antigens in influenza vaccines. Insect cell-derived VLPs expressing the N1 antigen of the 2009 pandemic H1N1 conferred a broader range of cross-protection than an inactivated split vaccine, which was highly strain-specific. Even in Fc receptor γ-knock out mice, N1 VLP immune sera conferred protection against homologous and heterologous challenge infections [21]. In another study, the NA antigens of A/California/04/2009 (H1N1) and A/Perth/16/2009 (H3N2) were co-expressed on the surface of the M1 derived from A/Michigan/73/2015 influenza viruses. These were self-assembled into VLPs in Sf9 insect cells, and their immunization in mice resulted in protection against lethal challenge infections with H1N1 and H3N2 viruses [22]. The importance of NA in protection against multiple clades of influenza viruses was demonstrated using NA-expressing VLPs. H5 antigens were co-expressed with N1, N5, N6, or by themselves on insect-derived VLPs, and the protection elicited by these particles was investigated in BALB/c mice. Compared to H5-expressing VLPs alone, co-formulation with NA antigens enhanced protection. Consistent with the findings demonstrated through the concept of multi-antigenic vaccines, increasing the variety of NA expression on baculovirus-expressed VLPs substantially enhanced protection against H5N1, H3N2, and H1N1 influenza viruses [23]. Mammalian cells could also be used to generate VLPS expressing the HA and NA of either A/Hong Kong/4801/2014 (H3N2) or B/Phuket/3073/2013. When BALB/c mice were immunized intramuscularly with mammalian cell-derived VLPs that expressed equal portions of HA and NA, functional antibodies against both glycoproteins were induced [24].

Investigating the impact of the VLP vaccines on the aging population is also of concern, and a few research studies investigating this have found interesting results. VLP vaccines were proven to be highly efficacious in aged mice with co-morbidities. Specifically, compared to the mice immunized with the inactivated H1N1 influenza virus, those immunized with the plant-derived H1-expressing VLPs induced greater CD4^+^ and CD8^+^ T cell responses and were generally better protected [25]. Immunization routes can affect the immune response induction in vaccinees. The impact of these multi-route immunization strategies was investigated by Hodgins et al. [26] using plant-derived VLP vaccines expressing the HA of A/California/07/2009 (pdmH1N1) in aged mice. Here, the authors compared the protection elicited in elderly mice when the VLPs were administered via three different strategies: prime-boost with intramuscular routes, a prime-pull strategy using intramuscular prime followed by intranasal boost, and a multi-modal route where VLP vaccines are simultaneously administered via both routes. The protection elicited by these three regimens was compared to that of split inactivated influenza vaccine control groups. Overall, survival rates were generally similar across all immunization groups, but VLP groups generally elicited greater splenic T cell responses and incurred less pulmonary inflammation and lesser lung virus titers compared to the split vaccines. Recently, while investigating the vaccine-induced cellular immune responses in mice immunized with this plant-derived H1 VLPs, a unique set of IL-1R1^+^ regulatory T cell populations was identified in the lungs of mice [27]. Although the exact mechanism of action has yet to be revealed, these cell populations may be integral for inhibiting pulmonary inflammation.

Multi-antigenic VLP vaccines are efficacious against several different strains of influenza viruses. Resultantly, studies examining the protection induced by these bivalent vaccines are prevalent. Baculovirus-derived VLPs expressing the H5 and H7 antigens of avian influenza viruses are one such example. A single dose of this VLP vaccine was sufficient to mount protection in immunized chickens against the highly pathogenic avian influenza virus challenge. Compared to the commercialized trivalent inactivated vaccines, the bivalent VLP vaccine incurred lesser pulmonary pathologies, and much of the lesions and inflammatory responses were alleviated [28]. One study compared the range of protection elicited by both mammalian cell-derived VLPs and protein subunit vaccines. Compared to mice immunized with the H5 and N6 subunits, neutralizing antibody titers were much higher in H5N6 VLP-immunized mice. The neutralizing potential of these sera was tested against pseudoviruses expressing H5 and a wide array of NA antigens. In almost all cases, irrespective of H5N2, H5N6, or H5N8 subtypes, neutralizing activity was greater in the sera acquired from the H5N6 VLP-immunized mice [29]. Insect cell-derived VLPs expressing the HA antigens of multiple clades were demonstrated to be more efficacious than single antigens. Chickens immunized once with VLPs co-expressing the highly pathogenic avian influenza HA antigens of clades 2.3.2.1c and 2.3.4.4c on a single VLP were better protected than VLPs expressing the HA of a single clade. The antibody responses against both clades were maintained for months, and the potential for scaling this VLP production at a low cost seemed to be a promising approach [30].

Studies investigating VLP vaccine-induced protection against influenza B viruses are severely lacking. Because of the emerging influenza B virus-borne infectious cases, VLP vaccine efficacies were evaluated in mice. HA and NA antigens derived from B/Washing/02/2019 Victoria lineage and B/Phuket/3073/2013 Yamagata lineage were used to generate VLPs in Sf9 cells, and these were intramuscularly administered into mice. Interestingly, none of the immunized mice perished upon challenge infection with a lethal dose of B/Colorado/06/2017 Victoria lineage virus, thus signifying the presence of VLP-induced cross-protection against mismatched B virus infections [31].

### 2.3. Improving the Efficacy of VLPs Vaccines

Although influenza vaccines have been available since the 20th century, they were incapable of preventing viral mutations, and seasonal influenza vaccines remain largely ineffective at times as the strain used in the vaccines failed to match those circulating the world. For this reason, developing a universal vaccine is paramount. Interestingly, the vaccine efficacy of the VLPs displaying the HA antigen of A/California/04/2009 was strikingly different from those of split vaccines. Immunized mice, either with the baculovirus-derived VLPs or split vaccines, were challenge infected with the homologous virus or a heterosubtypic virus A/Philippines/2/82/(H3N2) virus. While VLPs induced better protection against homologous challenge infection, heterosubtypic protection was noticeably higher in mice immunized with the split vaccines [32].

Computationally optimized broadly reactive antigen (COBRA) technology is being explored for influenza vaccine development. The potential for these COBRA-based antigen constructs was demonstrated using ferrets with pre-existing immunity to the H3N2 influenza virus. Here, the mammalian 293T cell-expressed COBRA H3 VLP vaccines conferred broad protection against both the historic vaccine strain and the multiple variants that were co-circulating simultaneously. Furthermore, the COBRA H3 VLPs also elicited greater hemagglutinin inhibition (HAI) and neutralizing antibody titers than VLPs expressing the wild-type H3 antigen [33]. Similar findings were also demonstrated from next-generation mammalian cell-derived COBRA VLP vaccines displaying the H3 antigen, most of which outperformed the wild-type H3 vaccines against historic and co-circulating variants [34]. COBRA technology was also utilized to develop avian influenza virus vaccines. In stark contrast to the VLPs expressing the HA antigen derived from A/whooper swan/Mongolia/244/2005, the 293T cell-expressed COBRA-2 VLP displaying the H5 sequence from the 2005–2006 period elicited greater immune responses. Furthermore, while the former failed to confer protection against antigenic drift variant A/duck/Vietnam/NCVD-672-2011, COBRA-2 HA VLP conferred protection in 80% of the immunized chickens [35].

Heterologous immunization is an interesting concept, and this regimen can improve the efficacy of vaccines. Live-attenuated influenza virus vaccine efficacy could be moderately improved by boosting with baculovirus-expressed VLPs containing the five tandem repeats of the M2e antigen (M2e5x) derived from human, swine, and avian influenza viruses. This immunization regimen enhanced protection against both A/Vietnam/rgH5N1 and A/PR8/H1N1 lethal challenge infections in immunized mice [36]. This M2e5x VLPs, when co-administered along with the influenza split vaccine via microneedle patch into mice, conferred better protection than the split vaccine alone and substantially enhanced cross-protection in mice [37].

Because the M2e5x VLPs were weakly immunogenic by themselves, methods to improve their protective efficacy have been explored. BALB/c mice immunized with the Sf9 cell-derived VLPs co-displaying the HA and M2e5x antigens were better protected against reassortant H5N1 avian influenza virus challenge infection than VLPs solely expressing the M2e5x [38]. Using the SpyTag/SpyCatcher technology, another study generated norovirus-like particles based on the VP1 capsid proteins co-displaying the M2e and the HA2 subunit derived from A/Puerto Rico/8/34 influenza virus to investigate their efficacy. While HA2-expressing VLPs induced virus-specific antibody responses, M2e VLPs failed to elicit detectable levels of virus-specific antibodies. Surprisingly, the HA2 VLP-induced antibodies failed to neutralize the influenza virus in vitro [39]. In one study, the M2e5x VLPs were generated using cellulose acetate phthalate dispersion technology. When these VLP were accompanied by Alhydrogel and monophosphoryl lipid A (MPL-A) adjuvants, they were capable of stimulating dendritic cells via the Th1 pathway [40]. Transdermally immunizing mice with the identical adjuvanted M2e5x VLPs elicited virus-specific antibody responses and triggered the proliferation of splenic T cells [41]. Compared to unadjuvanted M2e5x VLPs, the same adjuvanted VLP demonstrated significantly higher antigen presentation. This VLP vaccine was used to immunize mice through the epidermis via an ablative laser, which induced immune cell stimulation in immune organs [42]. One study used the M2e tandem repeat VLPs co-expressing the codon-optimized N1, N2, and influenza B NA consensus sequences. Immunizing mice with these multi-antigenic baculovirus-derived VLPs conferred protection against numerous influenza A viruses as well as both influenza B lineages. Surprisingly, aged mice immunized with these VLPs were also protected [43].

Incorporating adjuvants to improve VLP efficacy is another strategy. For example, both B-cell activating factor (BAFF) and a proliferation-inducing ligand (APRIL) are tumor necrosis factor (TNF) family ligands that could be used as molecular adjuvants. Genetically engineering baculovirus-derived VLPs to incorporate these molecular adjuvants can improve the protection induced by influenza VLPs. Using this approach, one study reported that fusing BAFF or APRIL to the transmembrane domain of the HA antigens can be an effective inducer of protective immunity against influenza virus challenge infections [44]. Incorporating interleukin-12 into influenza VLPs can also enhance protection against heterotypic influenza virus challenge infection. By anchoring IL-12 to the membranes of Sf9 cell-derived VLPs expressing the HA of A/tufted duck/Fukushima/16/2011 (H5N1) and A/Anhui/1/2013 (H7N9) avian influenza, immunized mice were protected against homologous challenge infection, but partial protection was observed against heterosubtypic challenge with A/PR/8/34 (H1N1) strain [45].

### 2.4. Alternative Strategies of Influenza VLP Use and Potential Improvement

VLP usage is not strictly limited to antigen delivery or vaccination purposes. These nanoparticles can also be utilized to understand the carefully orchestrated internal processing of influenza antigens in cells, as demonstrated using human monocyte-derived macrophages [46]. VLPs by themselves could also be used as adjuvants to current FDA-approved vaccines. In one study, bacterial flagellin molecules were incorporated into VLPs, which were then used as an adjuvant for the influenza split vaccines. By combining the flagellin-expressing VLPs with the split vaccine, Th1-biased antibody responses were induced in both wild-type and CD4-deficient transgenic mice [47]. A hybrid VLP based on HBc and flagellin was recently modified to express four tandem repeats of the influenza M2e in the surface-exposed D3 domain of the flagellin molecule. Mice immunized with these modified insect-derived VLPs were better protected than those immunized with the flagellin-HBc M2e VLPs with unmodified D3 region. Replacing the D3 domain with the M2e antigens further enhanced the survival of immunized mice following challenge infection with the A/Puerto Rico/8/34 (H1N1), A/California/09/2009 (H1N1pdm09), and A/Philippines/2/82 (H3N2) viruses [48].

Mutations can be introduced to improve the efficacy of influenza vaccines. By disabling the sialic acid receptor-binding activity of the HA antigen in A/California/07/09 (H1N1) expressed on VLPs, a noticeable increase in vaccine-induced protection was detected in mice. Specifically, antibody responses induced by these plant-derived HA VLPs were much more potent than the wild-type HA-expressing VLPs. Receptor-binding inhibited HA VLPs also incurred less pulmonary inflammation and further reduced the lung virus titer by two log orders lower than the HA wild-type VLPs [49].

Although VLP vaccines are still undergoing clinical evaluation, several strategies to improve VLP manufacturing have been documented. One such strategy is the improvements to down-stream processing of the VLPs. A proof-of-concept study demonstrated that an optimized membrane-based down-stream process could be crucial for influenza VLP vaccine production, especially when looking from an industrial perspective. An optimized membrane-based purification approach enabled approximately 80% product recovery while substantially minimizing production costs and time [50]. Dissolved oxygen (DO) is a key component for obtaining high VLP yields. Maintaining the DO at sufficiently high levels in a bioreactor setting was reported to improve the HA titer of H7N9 VLPs by 128-fold [51]. Insect cell-derived M2 VLPs were recently reported to possess inherent issues that hamper their production and final yield. M2 ion channel activity has been reported to be detrimental to VLP production in Sf9 cells, as they incurred cytopathological effects that affected the recombinant baculoviral replication and influenza protein expressions to ultimately reduce the total VLP yield. However, when the M2 inhibitor amantadine was present, there was a substantial increase in the expression of all antigen expressions. HA, M1, and M2 expressions on these VLPs were enhanced sevenfold, threefold, and threefold, each, respectively [52].

Developing a universal influenza vaccine is the ultimate goal for influenza vaccinologists, and one possible solution to this is the induction of broadly neutralizing antibodies (bNAbs), which are capable of binding to almost all HA variants. The presence of a highly conserved antibody binding site in the stem region revealed the feasibility of developing such a vaccine, but this was proven to be strikingly challenging. In short, simply removing the rapidly mutating HA domain that overcrowded the viral surface was difficult [53]. However, this was overcome using the novel ferritin-based nanoparticle. Immunization with this vaccine raised neutralizing antibody responses against the conserved stem region as well as the receptor binding site on the HA head domain. Antibodies induced by this vaccine neutralized H1N1 influenza variants from the years 1934–2007, as well as conferring protection in ferrets [54]. Following this approach, numerous studies involving ferritin-based nanoparticle vaccines are currently ongoing, which also reported heterologous and heterosubtypic protection [55,56]. These headless HA nanoparticle vaccines were strongly efficacious against a wide array of H1 and H3 variants in non-human primates, thereby signifying their developmental potential as universal influenza vaccines [57].

### 2.5. Influenza VLP Vaccines at the Clinical Level

VLP-based influenza vaccines have been ongoing for more than a decade, and the general trend seems to be that they are well-tolerated in vaccinees. Several clinical trials have been conducted for both avian and human influenza viruses. Plant-derived avian H5N1 VLPs were utilized in Phase 1 clinical trials in healthy adults 18–60 years of age (ClinicalTrials.gov: NCT00984945). In this trial, vaccines of all tested doses were well-tolerated in vaccinees with mild to self-limiting adverse events [58]. Another avian influenza VLP clinical trial (ClinicalTrials.gov: NCT00519389) reported that insect cell-derived VLPs expressing the HA, NA, and M1 proteins of H5N1 A/Indonesia/05/2005 (clade 2.1) induced antibody responses that preferentially bind to the oligomeric form of HA and the C terminus of NA, but the dose-sparing effect was not observed [59]. H7N9 VLP studies also underwent clinical trials. The role of saponin-based ISCOMATRIX adjuvant played a crucial role in enhancing HAI seroconversion and N9-inhibiting antibody responses [60,61].

Clinical trials were also conducted for the A/California/04/2009 (H1N1) pandemic influenza. Interestingly, when using bacterially produced VLPs to immunize human subjects in a Phase I clinical trial, non-adjuvanted VLPs conferred a similar level of immunogenicity and safety profile comparable to that of commercialized vaccines, whereas adjuvanted vaccines failed to meet the pre-determined immunogenicity endpoint [62]. Here, VLPs derived from Sf9 cells elicited a robust seroprotection rate even with a single immunization in participants [63]. The insect cell-based VLPs induced durable antibody response in vaccinees, which persisted up to 24 months post-immunization in human subjects [64].

Seasonal influenza VLP vaccines were also subjected to clinical evaluation, as tabulated below (Table 1). In the Phase 1/2 clinical trial (ClinicalTrials.gov: NCT01991587), quadrivalent VLPs produced in *Nicotiana benthamiana* by Medicago, Inc. induced a substantial increase in cross-reactive antibody responses that lasted for months [65]. These quadrivalent VLPs were further tested in two groups: healthy adults 18–49 of age and participants between the ages 50–64 (ClinicalTrials.gov: NCT02233816, NCT02236052). In both groups, immunizing participants with 30 μg of these plant-derived VLPs induced strong humoral and cellular immune responses against both homologous and heterologous strains. Furthermore, these VLPs were safe and well-tolerated in participants with minimum adverse events [66]. Recently, the large-scale randomized Phase 3 clinical trial results for these quadrivalent VLPs were published, which involved participants aged 18–64 and 65 years or older. Unfortunately, the VLP vaccines failed to meet the pre-determined primary endpoint of 70% absolute vaccine efficacy. Nonetheless, severe side effects were not detected, and the protection was similar to those of commercially available vaccines [67]. In another Phase 3 clinical trial, safety and immunogenicity profiles of the identical quadrivalent VLP vaccines were reported to be consistent with previous findings, and the results also demonstrated consistent manufacturing capability, which is crucial for novel platforms such as the VLPs [68]. Phase 3 clinical trial results for the quadrivalent VLP vaccine manufactured by Novavax were recently published. Although the VLPs induced humoral and cellular immune responses and were well-tolerated in older adults, clinical vaccine efficacies were not evaluated in this study [69]. Despite these limitations, the aforementioned studies demonstrated that VLPs are a promising alternative to traditional egg-based vaccines. Importantly, the universal influenza vaccine candidate ferritin nanoparticle was also successful in its Phase 1 clinical trial as individuals intramuscularly immunized with the H2-ferritin nanoparticles induced neutralizing antibodies against both H1 and avian H5 influenza viruses (ClinicalTrials.gov: NCT03186781) [70].

## 3. Respiratory Syncytial Virus (RSV)

### 3.1. Brief History of RSV, Vaccine Development, and Candidate Antigens

RSV is an antisense single-stranded RNA virus, which is phylogenetically categorized under the family Pneumoviridae, genus *Orthopneumovirus*. Structurally, the virus consists of 10 genes that encode in order the non-structural (NS) proteins NS1, NS2, nucleoprotein (N), polymerase co-factor (P), matrix protein (M), small hydrophobic (SH) protein, attachment glycoprotein (G), fusion glycoprotein (F), transcription processing factors M2-1, M2-2, and the large polymerase subunit (L) [71]. Among them, only the F, G, and SH antigens are expressed on the virus surface. While many of these antigens have been utilized as vaccine components, the most successful RSV vaccines at both preclinical and clinical levels were those expressing either F and/or G proteins. RSV is currently the main cause of lower respiratory infection in infants. Estimates revealed that on a global scale, RSV accounts for approximately 3 million hospitalizations and 60,000 deaths for children under the age of 5 [72]. The virus is not only a threat to infants but also to adults with comorbidities as well, especially individuals with chronic cardiopulmonary diseases [73]. Furthermore, most recent meta-analyses revealed that RSV-related case fatality proportions for individuals over 60 years of age residing in developed countries was approximately 8% [74], with an estimated 33,000 in-hospital deaths due to acute RSV infection [75].

RSV was first isolated in 1955 at Walter Reed Army Institute of Research in the United States from chimpanzees that were suffering from cold and coryza [76]. The isolated virus was initially named chimpanzee coryza agent (CCA), but its connection with human respiratory illness was unknown at the time. In the following year, Chanock et al. [77] isolated viral specimens from two children, which were indistinguishable from the CCA. Consequently, nomenclature changes were introduced, and the CCA, which was demonstrated to be capable of infecting humans, became known as the “respiratory syncytial virus,” as this was more fitting. Despite decades of global efforts, safe and efficacious RSV vaccine remains unavailable to this day. In the mid-1960s, there was a massive clinical trial failure that led to the death of two infants who were immunized with the infamous “Lot 100” formalin-inactivated RSV (FI-RSV). The truly paradoxical aspect was that the vaccines, which were meant to be protective, not only failed to elicit protection in vaccinees but also exacerbated the disease symptoms [78]. Given the rising safety concerns, RSV vaccine development has cautiously progressed, and the nature of vaccine-enhanced disease issue remain unresolved. Although VLPs are still under development, the outlook for these highly immunogenic particles is bright as these immunogenic particle vaccines could become a solution to the vaccine-associated enhanced respiratory disease (VAERD) that killed two infants in the 1960s, thus paving the path to commercially available RSV vaccines.

### 3.2. Pre-Clinical VLP Vaccine-Induced Protection and Novel Approaches

Research using VLP-based vaccines against RSV infections has only begun fairly recently. The first report of chimeric VLP construct expressing an RSV antigen was described in 2004 by Harvey et al. [79], whose work demonstrated that VLPs expressing the RSV M2 protein are capable of inducing CD8^+^ T cells ex vivo, but the protective efficacy of these self-assembled VLPs against RSV was not investigated. The first VLP study to investigate this aspect was done using the Newcastle disease virus (NDV) VLPs that expressed the ectodomain of RSV glycoprotein (G) fused to the transmembrane and cytoplasmic domains of NDV proteins. VLP constructs based on this method incurred substantially less inflammatory response and pathological damage than the notorious formalin-inactivated VLPs [80]. Using an identical approach, VLPs expressing the NDV-fused RSV fusion (F) protein and the G protein ectodomains were reported to confer robust and protective immune responses [81,82]. Epitope fractions are of considerable interest for vaccine antigens. For instance, a component of the F protein can also be used as an antigen. In one study, the well-characterized 24 amino acid epitope on the F protein was used as an antigen and assembled into VLPs along with the woodchuck hepadnavirus core protein. This epitope, which is identical to the palivizumab epitope, was reported to confer high titers of RSV-neutralizing antibody responses [83]. Following this trend, numerous chimeric VLP studies have been conducted to date that revolved around expressing RSV antigens on different virus backbones. For example, multiple studies have evaluated the protective efficacy of VLPs expressing the RSV surface glycoproteins on the influenza M1 protein [84,85]. P22 bacteriophage-derived VLPs encapsulating both RSV M and M2 proteins were reported to elicit T-cell responses against both antigens in mice [86]. Mammalian cell-derived VLPs that more closely resemble RSV by expressing the RSV M protein, F, and G antigens have also been reported. VLPs produced this way induced protection in both upper and lower respiratory tracts of the immunized cotton rats [87].

Recently, the importance of the pre-fusogenic (PreF) form of RSV F protein has been reported [88]. Since then, a massive number of studies exploring the protective efficacy of these PreF antigens using the VLP platform have significantly increased with consistent results. Using a human metapneumovirus matrix protein as the structural core, protective efficacies of the VLP constructs expressing either PreF or post-fusogenic (PostF) forms of the RSV F protein were compared. Surprisingly, those displaying both pre- and post-fusogenic F protein induced the highest level of neutralizing antibody responses [89,90]. PreF antigens are also much better inducers of neutralizing antibodies than the PostF forms of the RSV F protein. In one study, NDV-based chimeric VLP vaccine-induced immunities differed between RSV-primed and naïve mice. Specifically, the sheer amounts of neutralizing antibodies induced by PreF VLPs were much higher in the former of the two. The PostF VLPs, on the other hand, failed to activate high titers of neutralizing antibody responses in RSV-primed mice [91]. The protective efficacy of the identical VLPs expressing the stabilized PreF form of the RSV has been investigated in cotton rats, which resulted in potent neutralizing antibody responses with no enhanced respiratory disease [92].

As with baculovirus-derived influenza virus VLPs, multi-antigenic VLPs for RSV have been under development. VLP constructs displaying the tandem repeats of the RSV G protein (Gt) on the surface of influenza M1 protein were evaluated in murine models [93]. These RSV Gt VLPs ensured that the immunized mice experienced less pulmonary inflammation and reduced the viral lung loads following challenge infection. In a follow-up study to this work, the Gt antigens were paired with the PreF antigens, and the protection elicited by these VLPs was investigated. Co-formulating PreF and Gt antigens into VLPs conferred better protection than VLPs expressing either antigen alone. The severity of pulmonary inflammation and lung virus titers were lower in mice immunized with the PreF+Gt VLPs, albeit neutralizing antibody titers were comparable across the immunization groups [94]. Fusing multiple RSV antigen epitopes to the HBc also worked for VLP assembly. In one study, truncated fractions of HBc were fused with two epitopes from the RSV F antigen, a CTL epitope from the M2 antigen, and a truncated G protein fragment. This chimeric VLP, when intramuscularly administered into mice, induced Th1-biased immune responses while limiting Th2 cytokine production. These VLP vaccines also alleviated pulmonary inflammation and limited viral replication in the lungs of immunized mice [95]. Recently, an epicutaneous immunization approach to boost pre-existing immunity was demonstrated. Here, synthetic self-assembling lipopeptide VLPs termed “V-306,” which display multiple epitopes of the RSV F antigenic site II mimetic antigen, were constructed. When used as booster vaccines in mice, the vaccines were capable of protecting immunized mice by reducing the viral load without incurring VAERDs [96].

While F and G protein-expressing VLPs were extensively researched, this was not the case for the SH antigen. Many of the SH protein vaccine studies were based on live-attenuated viruses [97,98]. Although a lipid-emulsified vaccine expressing the SH protein ectodomain was tested in clinical trials [99], not a single VLP vaccine displaying the SH antigen has been reported to date. As there is a paucity of studies investigating the protective efficacy of SH antigen-expressing VLPs, conducting these studies could be beneficial to understanding this antigen’s immunogenicity and developmental potential.

### 3.3. Maternal Immunity and VLP Vaccines

Maternal immunization to investigate the efficacy of RSV vaccines in infants is a potential immunization strategy, as increasing maternal immunity during pregnancy could extend the induced protection to newborns. So far, only a handful of pre-clinical studies have attempted to investigate the protection elicited by these VLP vaccines using a maternal immunization strategy. To test this, cotton rats were immunized with NDV-based VLPs expressing PreF or PostF antigen along with the RSV G protein. While VLPs expressing both F and G antigens strongly enhanced the pre-existing immunity against RSV in dams irrespective of the pre- or post-fusogenic forms, the boosting effect was significantly greater in dams immunized with the PreF VLPs. This protection was carried on to the pups, as indicated by the lessened lung inflammation following RSV challenge infection [100]. While protection was elicited in pups, this was only applicable to the first set of offspring from the first pregnancy in cotton rat dams. Despite the high level of anti-PreF antibodies and neutralizing antibody titers in the dams, protection elicited by the NDV-based RSV VLPs drastically declined in pups of the second pregnancy [101].

Recently, several different mutations were introduced to the PreF-stabilized RSV F protein, and the resulting differences were compared by expressing these different mutants on chimeric NDV VLPs. Interestingly, these alternative versions of the PreF mutants were reported to elicit different populations of RSV F-specific antibody responses [102]. Two of these variants, termed the UC-2 and UC-3, were of particular interest. When these alternative forms of UC-2 and UC-3 variants of PreF VLPs were used to immunize pregnant cotton rats, higher quantities of neutralizing antibody responses were elicited in comparison to the standard DS-Cav1 variant. These strong neutralizing antibodies were passed on to the pups of immunized dams and conferred robust protection. Specifically, upon challenge infection, lung RSV titers in pups of UC-2 and UC-3 F VLP-immunized dams were 6- and 40-fold less than those observed in pups of DS-Cav1 F VLP-immunized dams [103]. As evidenced by these studies, maternal immunization with mutant forms of PreF VLPs could bring about promising results, but whether this approach addresses the short-lived immune response remains to be unraveled.

### 3.4. Other Strategies to Improve RSV VLP Vaccine Efficacy

It is widely perceived than VLP-based vaccines are highly immunogenic, and the immune responses induced in animal models may far exceed those elicited by other vaccine platforms. Compared to DNA vaccines expressing the RSV F antigen, insect-derived VLPs expressing the identical antigen generally conferred better protection. Specifically, mice immunized with the RSV F DNA vaccines experienced weight loss and pulmonary inflammation that may have resulted from an influx of plasmacytoid dendritic cells, monocytes, and others [104]. However, there is always room for improvement. Introducing mutations to the antigen of interest is critical for improving VLP vaccine efficacy. This was further demonstrated by introducing additional mutations to the pre-fusion stabilized DS-Cav1. Deletion of fusion peptide, paired with multiple mutations in one or more furin cleavage sites, resulted in significantly enhanced antigenicity against the pre-fusion site Ø and the antigenic sites I and II-specific monoclonal antibodies. Compared to the DS-Cav1, mice immunized with the baculoviral VLPs displaying these mutant antigens induced higher RSV-specific antibody responses, neutralizing antibody titers, and lung viral clearance post-challenge infection [105]. Amplifying VLPs (AVLPs) are also interesting and could be a potential alternative to live-attenuated virus vaccines. Compared to the traditional VLPs, AVLPs are similar in terms of safety. The sole difference is their capability to continuously express the foreign gene of interest in cells, which implies a significantly lower dosage required to elicit immune responses in hosts. Using this approach, RSV F and the transcription factor M2-1 genes were inserted into the parainfluenza virus backbone to generate AVLPs. A single dose of these VLPs in mice was sufficient to mount RSV-specific antibody responses and inhibit lung virus replication [106]. The ferritin nanoparticle strategy was also recently employed for RSV vaccine development. Here, after glycan modifications were made to mask non-neutralizing epitopes, Pre-F subunits were fused to the ferritin nanoparticle. This vaccine elicited robust antigen-specific antibodies in both mice and non-human primates. Virus-neutralizing antibodies were induced even after 150 days post-immunization, which were even more potent than those elicited by DS-Cav1 [107].

### 3.5. RSV VLPs as a Potential Solution to VAERD and Clinical Application in the Pre-Immune Population

Though etiologically difficult to define as the mechanism of disease enhancements varies between different respiratory pathogens, RSV vaccine-associated enhanced respiratory diseases appear to be characterized by exuberant Th2 immune response in the lungs of primed individuals [108]. Surprisingly, multiple studies have revealed that VLP immunization is capable of suppressing VAERD. For example, when BALB/c mice were immunized with the soluble F subunit vaccine, Th2-biased immune responses and lung histopathologies were detected. Contrary to this, baculovirus-derived VLPs expressing the RSV F antigen elicited immune responses, which were predominantly of Th1 origin as indicated by IFN-γ responses, and VAERD was not observed [109]. The nature of VLP-induced VAERD suppression could be attributed to the Th1 polarization of the host’s immune system. When human monocyte-derived dendritic cells were stimulated with RSV G VLPs *in vitro*, Th1-dominant immunity was detected. Contrastingly, stimulating these cells with FI-RSV vaccines elicited a Th2/Th17-biased immune response with depreciated non-interferon antiviral immune responses [110]. Consistent with this notion, identical findings were reported in nasal wash samples of RSV-infected infants, which were characterized by Th2/Th17 dominance [111]. The detailed mechanism contributing to VLP-induced Th1 immunity is currently unknown, but it is speculated to involve Toll-like receptor 4 (TLR4) signaling. For example, RSV VLPs can be sensed by TLR4 in vitro, which upregulates Th1 cytokine expressions that are associated with protection [87]. This shift in immunity has important implications, even for the currently most promising PreF antigen. Immunizing mice with PreF subunit vaccines adjuvanted with the Th2-biased alum resulted in lung pathology characterized by pulmonary eosinophilia and mucus accumulation post-challenge infection with RSV line 19. However, lung pathologies were inhibited when the vaccine adjuvants were switched to a more Th1/Th2-balanced Advax-SM [112]. Based on the sheer quantities of experimental data supporting Th1-immunity induction by the RSV VLPs, which are highly immunogenic per se and do not require additional adjuvants, developing these VLPs as vaccines can yield promising results especially compared to subunit vaccines. Also, studies investigating the intricate interplay between the RSV VLPs with the TLR family that leads to this Th1-bias could further aid in RSV vaccine development targeting the pre-immune population. Currently, the VLP vaccine manufactured by Icosavax, Inc. is the only RSV VLP vaccine progressing in Phase 1 clinical trial (ClinicalTrials.gov: NCT05664334). While it remains to be determined whether or not these VLPs can prevent VAERD formation in humans, the quest for clinical RSV vaccine will continue.

## 4. SARS-CoV-2

### 4.1. Coronaviruses

Coronaviruses are enveloped positive-sense single-stranded RNA viruses that are phylogenetically categorized under the family Coronaviridae, which can be further subdivided into 4 genera: alpha, beta, gamma, and delta [113]. They are currently regarded as the largest RNA virus reported to date, whose genome size ranges from 27–32 kb [114]. Structurally, while the bulk of their genome encodes various non-structural proteins, all coronavirus genomes essentially encode the envelope (E), membrane (M), nucleocapsid (N), and spike (S) protein [115]. In the past two decades of the 21st century, several major outbreaks involving coronaviruses have occurred. These include the SARS-CoV outbreak originating from Hong Kong in 2003 [116], the Middle East Respiratory Syndrome (MERS) of 2012 [117], and the most recent SARS-CoV-2 coronavirus disease 2019 (COVID-19) pandemic that resulted in approximately 621 million infections and 6.5 million deaths throughout the world [118]. Among the four major proteins expressed by the SARS-CoV-2, the S protein is of considerable interest. The S protein can be subdivided into S1 and S2 subunits, but the former is held to greater importance as this portion contains the receptor binding domain (RBD) required for successful viral entry. Similar to the SARS-CoV, SARS-CoV-2 is thought to initiate the infection in humans by utilizing the angiotensin-converting enzyme 2 (ACE2) [119]. Given the COVID-19 pandemic spreading at an unprecedented speed, developing an efficacious vaccine is a global priority. The protective efficacy of VLP vaccines is documented to be superior to those of traditional protein subunit vaccines. Specifically, VLPs expressing the S protein RBD induced five times more neutralizing antibody responses in immunized mice in comparison to the subunit RBD vaccine. Furthermore, the RBD-expressing VLPs were also capable of conferring protection against multiple coronaviruses, including several SARS-CoV-2 variants [120]. In light of this finding, continuous efforts to develop VLP vaccines against SARS-CoV-2 are ongoing.

### 4.2. Protective Efficacy of the SARS-CoV-2 VLPs at the Preclinical Level

Construction of the first SARS-CoV-2 VLPs was reported in 2020 by Xu et al. [121], who demonstrated that the M, E, and S proteins are indispensable for efficient VLP assembly in mammalian cells. Since then, multiple studies have investigated the protective efficacy of SARS-CoV-2 VLP vaccines at the pre-clinical level. Using the SpyTag/SpyCatcher system, synthetic VLPs expressing the RBD of S protein were generated, which induced strong neutralizing antibody responses in several animal models [122]. As with other respiratory viruses, insect cells were also utilized for VLP assembly. Recombinant baculoviruses expressing the SARS-CoV-2 M and S proteins were used to construct VLPs in insect cells, and these unadjuvanted VLPs, when inoculated into Syrian gold hamsters, conferred protection against the SARS-CoV-2 B.1.1.7 variant by lessening the degree of pulmonary symptoms [123]. Importantly, as with other VLPs, SARS-CoV-2 VLPs do not necessarily need to be expressing structural components of native viruses. Rather, they can be produced using a wide array of cell lines or viruses to construct chimeric VLPs, and even under these conditions, the VLP vaccines continue to demonstrate profound protective efficacy in animal models. A 60-mer dodecahedric VLP derived from the human adenovirus, named “ADDomer,” was utilized to develop a unique VLP vaccine. Here, the surface of these ADDomers was decorated with SARS-CoV-2 S RBDs using the SpyTag/Catcher technology. This vaccination approach conferred a strong neutralizing response against the pseudo-typed virus, which far exceeded those elicited by the sera of convalescing patients [124]. Similar to the pre-fusion domain of RSV, the pre-fusogenic form of the SARS-CoV-2 S protein contains potent neutralizing activity. NDV-based VLPs expressing the prefusion-stabilized SARs-CoV-2 S protein evoked virus-neutralizing antibody responses in mice [125]. Immunizing mice with the AP205 bacteriophage-derived VLPs expressing the receptor binding motif (RBM) of the S protein RBD induced high titers of antigen-specific antibody responses. These VLP vaccines elicited strong IgG2a antibody responses required for protection and were capable of neutralizing SARS-CoV-2 [126].

Several studies have investigated the immunogenic effects of insect cell-derived chimeric SARS-CoV-2 VLP constructs. Influenza VLPs expressing the codon-optimized full-length S, S1, or S2 subunits were reported to induce virus-specific antibody responses in mice, but the neutralizing antibody responses were mediocre and failed to fully inhibit the virus receptor and ligand binding interaction [127]. Immunizing K18-hACE2 mice with HEK293T-derived chimeric influenza VLPs reduced the lung virus titers and promoted their survival post-challenge infection with SARS-CoV-2, albeit with moderate levels of pulmonary inflammation and brain lesion [128]. An interesting approach was recently reported. Using a similar approach, H5N1 avian influenza VLPs that express the codon-optimized matrix and SARS-CoV-2 S proteins were reported to induce both cellular and humoral immune responses in mice. Adjuvanting these baculoviral VLPs with CpG ensured that all of the immunized mice survived the challenge infection with minimum weight loss and a significant reduction in SARS-CoV-2 RNA copy numbers in the lungs [129].

Only a limited number of VLP vaccine efficacy studies were performed in non-human primates. Plant-derived VLPs expressing the SARS-CoV-2 S protein were proven to be immunogenic in rhesus macaques but supplementing these vaccines with the CpG adjuvants further enhanced both cellular and humoral immune responses. These findings were accompanied by a substantial reduction in viral titer and the absence of vaccine-associated enhanced disease [130]. Nearly identical findings were observed from rhesus macaques immunized with the AP205-derived capsid VLPs. Boost immunization with this VLP vaccine elicited a more than 50-fold increase in neutralizing antibody titers, significant lung virus titer reduction, and vaccine-enhanced disease in the lungs was undetected [131]. Overall, the protective efficacy of VLP vaccines at the pre-clinical level is outstanding, and as such, these platforms are currently being tested at the clinical level.

### 4.3. VLPs: How Do They Fare against the Emerging Variants of Concern (VOCs)?

As with all viruses, mutations in the genetic sequences of SARS-CoV-2 result in changes that can have diverse consequences in the viral properties, whether it be antigenicity, transmissibility, or others. Since the initial discovery of SARS-CoV-2 in late 2019, numerous variants possessing distinct mutations in the genetic sequences have emerged. The WHO has stated that one or more of the following three criteria must be met to be considered as VOCs: (1) increased transmissibility or negative changes to the COVID-19 epidemiology, (2) changes to clinical manifestation or virulence in hosts, or (3) reduced efficacy of vaccines and other prophylactic tools [132]. Currently, the Omicron variant (B.1.1.529) is the major circulating VOC, although four other previously circulating VOCs, such as the Alpha (B.1.1.7), Beta (B.1.351), Gamma (P.1), and Delta (B.1.617.2) variants are still being vigilantly monitored [132]. Given these circumstances, vaccines that can confer protection against these emerging variants are direly needed.

Only a small number of studies attempted to investigate the protective efficacy of VLPs against VOCs. In one study, a multi-component approach to VLP construction was employed. Here, the Sf9 insect cell-derived S1 subunit was conjugated to the AP205 bacteriophage via SpyTag/SpyCatcher system, and VLPs generated via this approach ensured potent antibody induction in immunized mice, which strongly neutralized the Wuhan (L008) and the UK (B.1.1.7) alpha variants of the SARS-CoV-2 [133]. Compared to the soluble RBD immunization, RBDs displayed on these capsid VLPs were more immunogenic and elicited higher anti-S antibody responses in mice, and a single dose of this vaccine was sufficient to confer a comparable level of neutralizing titer to those from convalescent patients [134]. In another study, genetically grafting the RBM of SARS-CoV-2 on the surface of cucumber mosaic virus (CCMV) that was immunogenically optimized via incorporation of tetanus toxin-derived helper T cell epitope induced immune responses in mice and rabbits. More importantly, these RBM-expressing VLPs demonstrated cross-reactivity with the RBDs of multiple variants containing mutations in the RBD [135]. Further studies involving these CCMV VLPs were conducted to evaluate the protective efficacy of these vaccines. Here, Chang et al. [136] fused the epitopes of SARS-CoV-2 RBM and the SARS-CoV-2 fusion peptide in the S2 domain with the CCMV subunits to construct a novel double mosaic VLP that elicited high levels of virus-neutralizing antibody responses. To address the problems posed by the VOCs, the same research group also tested whether these VLP vaccines could be effective against multiple emerging variants of SARS-CoV-2. The wild-type RBD expressed on these VLPs were capable of interacting with the Beta, Gamma, and Delta VOCs with high avidities [137].

### 4.4. Potential Application of VLPs to Study the Role of Mutations and Alternative Uses of SARS-CoV-2 VLP Vaccines

Continuous mutations and evolving viruses pose a tremendous challenge to vaccine development. However, VLPs can be used to investigate how specific mutations can alter the protective efficacy of vaccines. In one study, by deliberately introducing mutations resembling those of Omicron or Delta SARS-CoV-2 variants, the authors delineated that mutations in any of the key structural components can have a large impact on the viral particle assembly and their neutralization. Surprisingly, using Omicron-mutated SARS-CoV-2 VLPs, it was revealed that the Omicron variants are 15-fold more likely to evade neutralizing antibody responses in convalescing patients and vaccinated individuals [138]. Recently, Servellita et al. [139] conducted VLPs and live virus-based neutralization assays using clinical samples. The authors of this study revealed that in addition to the waning antibody responses in vaccinees, the protection conferred by these virus-neutralizing antibody responses was substantially reduced against Delta and Omicron variants. Furthermore, upon a breakthrough infection with either of the two variants, there was diminished variant-specific cross-neutralizing activity. Moreover, VLPs can also be utilized for SARS-CoV-2 diagnostic purposes, specifically as positive controls. The VLPs can be constructed as SARS-CoV-2 RNA transcript-encapsidating nanoparticle carriers, and the feasibility of this approach was validated using plant-derived CCMV and Qβ phage payload modules [140].

With the SARS-CoV-2 becoming more of a seasonal respiratory disease, as in the case of influenza viruses, continued measures to improve SARS-CoV-2 vaccines are a necessity. To address this, developing vaccines that confer dual protection against both influenza and SARS-CoV-2 would be ideal. Glycosylphosphatidylinositol-anchored SARS-CoV-2 S RBD was fused to the GM-CSF adjuvant, and this was incorporated into influenza VLPs expressing the HA and M1 derived from influenza A/Puerto Rico/8/1934 (PR8). This mammalian cell-derived VLP vaccine conferred protection against both influenza and SARS-CoV-2 virus challenge infection in mice [141]. Incorporating cyclic GMP-AMP (cGAMP) into VLPs significantly increased the vaccine immunogenicity against multiple respiratory viruses. In one study, HIV-derived VLPs that were pre-loaded with cGAMP were used to demonstrate the importance of these immunostimulatory molecules. When influenza HA or the SARS-CoV-2 S antigens were expressed on these cGAMP-loaded VLPs, these vaccines induced greater antigen-specific antibody responses and virus-neutralizing antibody titers against respective viruses than the unloaded VLP counterparts [142]. Therefore, applying these concepts would enable the rational design of vaccines to aid in the ongoing combat against respiratory diseases.

### 4.5. Improvements to Current SARS-CoV-2 Vaccines

From an economical perspective, yeast-expressed VLPs would be the system of choice that meets the aforementioned production criteria. Genetically engineering SARS-CoV-2 RBD not only improved the VLP yields in the yeast production system but also enhanced the immunogenicity of the vaccines in mice. Furthermore, these SpyTag/SpyCatcher-based VLPs induced antibodies which also interacted with SARS-CoV-2 variants of concern and even prevented drastic weight loss in hamsters following challenge infection [143]. Specifically, hamsters immunized with the alum-adjuvanted VLPs expressing the engineered RBD of S protein lost 15% of their initial weight by 6 days post-infection (dpi). However, further adjuvanting this vaccine with CpG prevented bodyweight loss not exceeding approximately 7% [143]. Recently, the protective efficacy of VLP vaccines was compared to the FDA-approved Pfizer mRNA vaccines in K18-hACE2 transgenic mice, which were previously developed for lethal SARS-CoV infection studies. SpyTag/SpyCatcher-based VLP constructs expressing the RBD of SARS-CoV-2 on the surface of hepatitis B surface antigen protected these mice, and the protection induced by these vaccines was comparable to those of commercially available mRNA vaccines [144].

The VLPs currently under development could also be used along with the emergency-approved SARS-CoV-2 vaccines. Heterologously immunizing mice with plant-derived VLP vaccine along with the Moderna mRNA-1273 vaccine conferred a high level of virus-specific antibodies. The VLP vaccine used in this study efficiently boosted the B-cells that were primed with the RBD-expressing mRNA vaccines. The prime-boost regimen involving mRNA and VLPs was just as effective when the immunization orders were reversed, that is, priming with VLP and boosting with mRNA [145].

The efficacy of ferritin nanoparticles expressing the S protein of SARS-CoV-2 was recently investigated. A single immunization with these ferritin nanoparticles elicited virus-neutralizing antibody titers in mice, which were at least twofold greater than sera acquired from COVID-19 convalescing patients [146]. Another study reported similar findings. Spike-ferritin nanoparticle (SpFN) induced robust neutralizing antibody titers after a single immunization, which was comparable to responses acquired from RBD-fused ferritin nanoparticles after two immunizations. Furthermore, antibodies induced by the SARS-CoV-2 ferritin nanoparticle vaccine were also capable of neutralizing SARS-CoV-2, thus implying their broadly protective immunity induction [147].

### 4.6. Clinical Development and Approved SARS-CoV-2 VLP Vaccines

As with influenza and RSV, FDA-approved SARS-CoV-2 VLP vaccines are currently unavailable. Much of the VLP-based vaccine remains at the preclinical level, with several being evaluated in clinical trials as described elsewhere [148]. Here, we briefly summarize some of the SARS-CoV-2 VLPs currently being evaluated at the clinical level. LYB001 is a VLP vaccine expressing the RBD of SARS-CoV-2 S protein adjuvanted with alum. The vaccine is manufactured by Yantai Patronus Biotech Co., Ltd., based in China. This vaccine recently finished its Phase 1 clinical trial and is now undergoing Phase 2/3 trials. The participants are immunized thrice at 4-week intervals through the intramuscular route. VBI-2902a is an enveloped VLP vaccine manufactured by Variation Biotechnologies Inc., based in the United States. This vaccine expresses the modified pre-fusion stabilized S protein on the surface, adjuvanted with aluminum phosphate. The vaccine is being evaluated in Phase 1/2 clinical trials, which involves immunizing patients a total of two times at 28-day intervals through the intramuscular route. A SARS-CoV-2 VLP expressing all four of its structural proteins is being evaluated. The vaccine, though unnamed, is reported to be developed by the Scientific and Technological Research Council of Turkey and is undergoing a Phase 2 clinical trial. At the time of writing this manuscript, initial positive data were released for the VBI-2902a, but clinical data for the LYB001 and SARS-CoV-2 VLP either remain largely undisclosed or inaccessible.

The ABNCoV2 is a capsid VLP adjuvanted with the MF59. This vaccine expresses the S protein RBD, and vaccines are administered twice through the intramuscular route. The Phase 1 clinical trial for this vaccine was carried out by Radboud University Medical Center (ClinicalTrials.gov: NCT04839146) and is currently undergoing Phase 3 clinical evaluation in the United States. CoVLP is another VLP vaccine developed by Medicago Inc. and GlaxoSmithKline. In this VLP vaccine, the pre-fusion stabilized S glycoprotein was modified by replacing its transmembrane and cytoplasmic tail portion with that of HA from A/Indonesia/5/2005 (H5N1) to enhance VLP assembly. Participants were immunized twice with this plant-derived VLP with AS03 or CpG1018 adjuvants. In the Phase 1 trial (ClinicalTrials.gov: NCT04450004), co-formulating this VLP with AS03 induced neutralizing antibody titers that were 10-fold higher than those detected from convalescent sera [149]. In the Phase 3 clinical trial, the protective efficacy for the CoVLP+AS03 adjuvant formulation was approximately 70% against moderate to severe disease [150]. The Canadian government has officially approved the domestic use of this vaccine for clinical purposes. A brief list of SARS-CoV-2 vaccines listed under ClinicalTrials.gov is tabulated below (Table 2). Currently, a ferritin-based nanoparticle vaccine is also being evaluated at the clinical level (NCT04784767).

## 5. Methods of Improving VLP Stability

While vaccine-induced protection is important, it is worth noting that many of these efficacious vaccines remain unattainable for numerous countries. Therefore, developing a highly efficacious vaccine that can be scaled to high yields at a low cost is desirable. This is particularly the case for mRNA-based SARS-CoV-2 vaccines. From a structural perspective, RNA is unstable and prone to degradation. Consequently, mRNA vaccines require cold chain logistics and management, which are difficult in developing countries. Fortunately, VLPs could address these limitations. The temperature-stable aspect of VLP vaccines was demonstrated using Cervarix, an FDA-approved VLP vaccine against HPV, which was developed by GlaxoSmithKline. Here, the vaccines retained their stability and immunogenicity for at least 3 years when stored at 2–8 °C. Surprisingly, even when the cold chain conditions were broken by exposing the VLPs to 37 °C or 25 °C for weeks, the VLPs’ structural stability was retained [151]. Storing VLPs at optimal conditions is another factor contributing to VLP stability. In one study, a natural deep eutectic system comprising trehalose and glycerol was successfully used to retain the stability of HA-expressing VLPs, which were derived from the insect High Five cells. Under this condition, the physical integrity of the VLPs was retained for approximately 4 h when stored at 50 °C, and storing influenza VLPs at room temperature for over 1 month had no impact on VLP degradation or the HA titer [152]. SARS-CoV-2 RBM-expressing CCMV VLPs were also reported to be strikingly stable, as they retained their stability for 14 months when stored at 4 °C with no signs of degradation [135]. SARS-CoV-2 S RBD expressed on AP205 bacteriophages are currently undergoing clinical evaluation, and the temperature-dependent stability of these VLPs was recently tested, which demonstrated that these VLPs could be stored at ambient temperatures for months while retaining their stability. Specifically, lyophilizing these VLP vaccines ensured that the vaccines remained stable for up to 6 months at ambient temperature. Furthermore, lyophilizing pre-clinical influenza VLP vaccines displaying the HA stalk region also were not subjected to degradation when lyophilized or incubated at ambient temperatures for 2 months [153]. While a study investigating the temperature stability of VLPs for RSV remains unreported, they are anticipated to retain their stability, as demonstrated in the aforementioned studies.

Although the experimental findings described above highlight the temperature stability aspect of the VLPs, one must take into account that each VLP is unique and variations in structural properties are highly plausible. Consequently, directly translating or applying the findings observed from one VLP to draw a general conclusion would be misleading. For example, the FDA-approved Cervarix VLP vaccine underwent substantial downstream processes, which are time-consuming and costly from a manufacturing standpoint, that influenced the structural properties of the VLPs. As most of the experimental VLPs were not subjected to such purification steps to ensure their suitability for clinical use, the stability aspect of FDA-approved VLPs does not apply to those that were not subjected to identical conditions.

Several strategies have been developed to improve the mucosal immune response induced by the VLP vaccines. One such method is developing degradation-resistant VLPs that can endure the highly acidic environmental conditions of the stomach following oral immunization. This can be achieved by exploiting the structural components of gastrointestinal protozoan parasites such as *Giardia lamblia*. The cysteine-rich variant-specific surface proteins (VSPs) of *G. lamblia* not only serve as a proteolytic degradation-resilient tool but also act as TLR4-dependent immunostimulatory molecules. Using this concept, Serradell et al. [154] demonstrated that these VSP-pseudotyped VLPs expressing the HA and NA antigens of the influenza A virus conferred robust mucosal immune responses in immunized mice. Upon a lethal challenge infection with the mouse-adapted variant of the H5N1 influenza virus, the VSP-pseudotyped VLPs conferred near-complete protection, while VLPs lacking the *G. lamblia* VSP components failed to prevent weight loss or elicit 100% survival. This approach was also used for SARS-CoV-2 VLPs to prevent their degradation in the gastrointestinal tract, which successfully induced mucosal immune responses in mice and hamsters when administered through the oral route [155]. Because mucosal immunization induces greater immune responses in vaccinees than those immunized via parenteral routes, efforts for mucosal vaccine development should be continued, as doing so would also address issues arising from trypanophobia in children.

## 6. Conclusions

Influenza, RSV, and SARS-CoV-2, since their initial discovery, have placed a tremendous emphasis on the need for safe and efficacious vaccine development. With the recent advances in biotechnology enabling the construction of VLPs, massive improvements to the field of vaccinology have been met at unprecedented speed, and their development offered a more effective vaccination strategy against emerging respiratory pathogens. Existing evidence from copious amounts of pre-clinical data suggests that VLPs are effective prophylactic tools against these three viruses, and this fueled their further development in clinical trials. While none of the VLP vaccines described here are commercially available, with CoVLP being the sole exception, at least within Canada, we anticipate that VLP-based vaccines for the respiratory RNA viruses covered here will be available for clinical use within a few decades.

## Figures and Tables

**Table 1:** Influenza VLP vaccines and their clinical trials.

Target	Antigens and Virus Strain	Responsible Party	Clinical Trial Phase and Identifier
Avian	HA (A/Indonesia/05/05, H5N1)	IDRI	Phase 1 NCT01657929
Avian	HA (A/Indonesia/05/05, H5N1)	Novavax	Phase 1/2 NCT00519389, NCT01594320, NCT01596725
Avian	H7N9 (A/Anhui/1/13)	Novavax	Phase 1/2 NCT01897701, NCT02078674
Avian	HA (A/Indonesia/05/05, H5N1)	Medicago	Phase 2 NCT01991561
Avian	HA (A/Hanzhou/1/13, H7N9)	Medicago	Phase 1 NCT02022163
Trivalent Seasonal	HA (A/Brisbane/59/07, H1N1) HA (A/Brisbane/10/07, H3N2) HA (B/Brisbane/60/08, Victoria)	Novavax	Phase 2 NCT01014806
Quadrivalent Seasonal	HA (A/Brisbane, H1N1) HA (A/Kansas, H3N2) HA (B/Maryland, Victoria) HA (B/Phuket, Yamagata)	Novavax	Phase 3 NCT04120194
Quadrivalent Seasonal	HA (A/California/07/09, H1N1) HA (A/Victoria/361/11, H3N2) HA (B/Brisbane/60/08, Victoria) HA (B/Massachusetts/02/12, Yamagata)	Medicago	Phase 3 NCT03301051, NCT03321968, NCT03739112
Pandemic	HA (A/California/07/09, H1N1)	Novavax	Phase 2 NCT01072799
Universal	H1-Ferritin HA (A/New Caledonia/20/1999, H1N1)	National Institute of Allergy and Infectious Diseases (NIAID)	Phase 1 NCT03814720
Universal	H2-Ferritin HA (A/Singapore/1/1957, H2N2)	National Institute of Allergy and Infectious Diseases (NIAID)	Phase 1 NCT03186781
Universal	H10-Ferritin HA (A/Jianxi/IPB13/2013, H10N8)	National Institute of Allergy and Infectious Diseases (NIAID)	Phase 1 NCT04579250

**Table 2:** A list of SARS-CoV-2 VLP vaccines currently under clinical trial.

Vaccine	Antigens and Adjuvants	Manufacturer/Responsible Party	Clinical Trial Phase and Identifier
LYB001	S protein RBD; alum adjuvant	Yantai Patronus Biotech Co., Ltd.	Phase 2/3 NCT05125926, NCT05137444
VBI-2902a	Pre-fusion stabilized S protein; alum adjuvant	Variation Biotechnologies Inc.,	Phase 1/2 NCT04773665
SARS-CoV-2 VLP (unnamed)	All structural proteins of the SARS-CoV-2	Scientific and Technological Research Council of Turkey	Phase 2 NCT04962893
ABNCoV2	SARS-CoV-2 S RBD; MF49 adjuvant	Bavarian Nordic	Phase 3 NCT05329220
CoVLP	Modified pre-fusion stabilized S protein; AS03 adjuvant	Medicago Inc., and GlaxoSmithKline	Phase 3 NCT04636697
Spike-ferritin nanoparticle (SpFN)	Spike-ferritin; ALFQ adjuvant	U.S. Army Medical Research and Development Command	Phase 1 NCT04784767

## Data Availability

Not applicable.

## References

[1] GBD 2016 Lower Respiratory Infections Collaborators (2018). Estimates of the global, regional, and national morbidity, mortality, and aetiologies of lower respiratory infections in 195 countries, 1990–2016: A systematic analysis for the Global Burden of Disease Study 2016. Lancet Infect Dis..

[2] Siegel J.D., Rhinehart E., Jackson M., Chiarello L. (2007). 2007 Guideline for Isolation Precautions: Preventing Transmission of Infectious Agents in Health Care Settings. Am. J. Infect Control.

[3] Baker R.E., Mahmud A.S., Miller I.F., Rajeev M., Rasambainarivo F., Rice B.L., Takahashi S., Tatem A.J., Wagner C.E., Wang L.F. (2022). Infectious disease in an era of global change. Nat. Rev. Microbiol..

[4] Mercer A.J. (1985). Smallpox and epidemiological-demographic change in Europe: The role of vaccination. Popul. Stud..

[5] Burnet F.M. (1933). Specific Agglutination of Bacteriophage Particles. Br. J. Exp. Pathol..

[6] Hyman P., Trubl G., Abedon S.T. (2021). Virus-Like Particle: Evolving Meanings in Different Disciplines. Phage.

[7] Kushnir N., Streatfield S.J., Yusibov V. (2012). Virus-like particles as a highly efficient vaccine platform: Diversity of targets and production systems and advances in clinical development. Vaccine.

[8] Weis S., Te Velthuis A.J.W. (2021). Influenza Virus RNA Synthesis and the Innate Immune Response. Viruses.

[9] Tong S., Zhu X., Li Y., Shi M., Zhang J., Bourgeois M., Yang H., Chen X., Recuenco S., Gomez J. (2013). New world bats harbor diverse influenza A viruses. PLoS Pathog..

[10] Martin P.M., Martin-Granel E. (2006). 2500-year evolution of the term epidemic. Emerg. Infect Dis..

[11] Potter C.W. (2001). A history of influenza. J. Appl. Microbiol..

[12] Smyk J.M., Szydłowska N., Szulc W., Majewska A. (2022). Evolution of Influenza Viruses-Drug Resistance, Treatment Options, and Prospects. Int. J. Mol. Sci..

[13] Layton G.T., Harris S.J., Myhan J., West D., Gotch F., Hill-Perkins M., Cole J.S., Meyers N., Woodrow S., French T.J. (1996). Induction of single and dual cytotoxic T-lymphocyte responses to viral proteins in mice using recombinant hybrid Ty-virus-like particles. Immunology.

[14] Neirynck S., Deroo T., Saelens X., Vanlandschoot P., Jou W.M., Fiers W. (1999). A universal influenza A vaccine based on the extracellular domain of the M2 protein. Nat. Med..

[15] Watanabe T., Watanabe S., Neumann G., Kida H., Kawaoka Y. (2002). Immunogenicity and protective efficacy of replication-incompetent influenza virus-like particles. J. Virol..

[16] Latham T., Galarza J.M. (2001). Formation of wild-type and chimeric influenza virus-like particles following simultaneous expression of only four structural proteins. J. Virol..

[17] Ong H.K., Yong C.Y., Tan W.S., Yeap S.K., Omar A.R., Razak M.A., Ho K.L. (2019). An Influenza A Vaccine Based on the Extracellular Domain of Matrix 2 Protein Protects BALB/C Mice Against H1N1 and H3N2. Vaccines.

[18] Kirsteina A., Akopjana I., Bogans J., Lieknina I., Jansons J., Skrastina D., Kazaka T., Tars K., Isakova-Sivak I., Mezhenskaya D. (2020). Construction and Immunogenicity of a Novel Multivalent Vaccine Prototype Based on Conserved Influenza Virus Antigens. Vaccines.

[19] Li M., Guo P., Chen C., Feng H., Zhang W., Gu C., Wen G., Rao V.B., Tao P. (2021). Bacteriophage T4 Vaccine Platform for Next-Generation Influenza Vaccine Development. Front. Immunol..

[20] Sharma J., Shepardson K., Johns L.L., Wellham J., Avera J., Schwarz B., Rynda-Apple A., Douglas T. (2020). A Self-Adjuvanted, Modular, Antigenic VLP for Rapid Response to Influenza Virus Variability. ACS Appl. Mater. Interfaces.

[21] Kim K.H., Lee Y.T., Park S., Jung Y.J., Lee Y., Ko E.J., Kim Y.J., Li X., Kang S.M. (2019). Neuraminidase expressing virus-like particle vaccine provides effective cross protection against influenza virus. Virology.

[22] Menne Z., Pliasas V.C., Compans R.W., Glover S., Kyriakis C.S., Skountzou I. (2021). Bivalent vaccination with NA1 and NA2 neuraminidase virus-like particles is protective against challenge with H1N1 and H3N2 influenza A viruses in a murine model. Virology.

[23] Kang H.J., Chu K.B., Yoon K.W., Eom G.D., Mao J., Kim M.J., Lee S.H., Moon E.K., Quan F.S. (2022). Multiple Neuraminidase Containing Influenza Virus-like Particle Vaccines Protect Mice from Avian and Human Influenza Virus Infection. Viruses.

[24] Buffin S., Peubez I., Barrière F., Nicolaï M.C., Tapia T., Dhir V., Forma E., Sève N., Legastelois I. (2019). Influenza A and B virus-like particles produced in mammalian cells are highly immunogenic and induce functional antibodies. Vaccine.

[25] Hodgins B., Pillet S., Landry N., Ward B.J. (2019). A plant-derived VLP influenza vaccine elicits a balanced immune response even in very old mice with co-morbidities. PLoS ONE.

[26] Hodgins B., Pillet S., Landry N., Ward B.J. (2019). Prime-pull vaccination with a plant-derived virus-like particle influenza vaccine elicits a broad immune response and protects aged mice from death and frailty after challenge. Immun. Ageing.

[27] Alvarez F., Istomine R., Hendin H., Hodgins B., Pillet S., Fritz J.H., Charland N., Ward B.J., Piccirillo C.A. (2022). A Hemagglutinin 1 Carrying Plant-Based Virus-Like Particle Vaccine Generates an Efficacious Cellular Response by Exploiting IL-1 Signaling in Both Adult and Aged Mice. Immunohorizons.

[28] Hu J., Peng P., Li J., Zhang Q., Li R., Wang X., Gu M., Hu Z., Hu S., Liu X. (2021). Single Dose of Bivalent H5 and H7 Influenza Virus-Like Particle Protects Chickens Against Highly Pathogenic H5N1 and H7N9 Avian Influenza Viruses. Front. Vet. Sci..

[29] Yang Y.H., Tai C.H., Cheng D., Wang Y.F., Wang J.R. (2022). Investigation of Avian Influenza H5N6 Virus-like Particles as a Broad-Spectrum Vaccine Candidate against H5Nx Viruses. Viruses.

[30] Kang Y.M., Cho H.K., Kim J.H., Lee S.J., Park S.J., Kim D.Y., Kim S.Y., Park J.W., Lee M.H., Kim M.C. (2021). Single dose of multi-clade virus-like particle vaccine protects chickens against clade 2.3.2.1 and clade 2.3.4.4 highly pathogenic avian influenza viruses. Sci. Rep..

[31] Kang H.J., Chu K.B., Yoon K.W., Eom G.D., Mao J., Quan F.S. (2022). Cross-Protection Induced by Virus-like Particles Derived from the Influenza B Virus. Biomedicines.

[32] Lee G.J., Chu K.B., Inn K.S., Moon E.K., Quan F.S. (2020). Vaccine Efficacy Induced by 2009 Pandemic H1N1 Virus-Like Particles Differs from that Induced by Split Influenza Virus. Immunol. Investig..

[33] Allen J.D., Jang H., DiNapoli J., Kleanthous H., Ross T.M. (2019). Elicitation of Protective Antibodies against 20 Years of Future H3N2 Cocirculating Influenza Virus Variants in Ferrets Preimmune to Historical H3N2 Influenza Viruses. J. Virol..

[34] Allen J.D., Ross T.M. (2021). Next generation methodology for updating HA vaccines against emerging human seasonal influenza A(H3N2) viruses. Sci. Rep..

[35] Ross T.M., DiNapoli J., Giel-Moloney M., Bloom C.E., Bertran K., Balzli C., Strugnell T., Mariana S.E.S., Mebatsion T., Bublot M. (2019). A computationally designed H5 antigen shows immunological breadth of coverage and protects against drifting avian strains. Vaccine.

[36] Lee Y.T., Kim K.H., Ko E.J., Kim M.C., Lee Y.N., Hwang H.S., Lee Y., Jung Y.J., Kim Y.J., Santos J. (2019). Enhancing the cross protective efficacy of live attenuated influenza virus vaccine by supplemented vaccination with M2 ectodomain virus-like particles. Virology.

[37] Kim M.C., Kim K.H., Lee J.W., Lee Y.N., Choi H.J., Jung Y.J., Kim Y.J., Compans R.W., Prausnitz M.R., Kang S.M. (2019). Co-Delivery of M2e Virus-Like Particles with Influenza Split Vaccine to the Skin Using Microneedles Enhances the Efficacy of Cross Protection. Pharmaceutics.

[38] Kang H.J., Chu K.B., Lee D.H., Lee S.H., Park B.R., Kim M.C., Kang S.M., Quan F.S. (2019). Influenza M2 virus-like particle vaccination enhances protection in combination with avian influenza HA VLPs. PLoS ONE.

[39] Heinimäki S., Lampinen V., Tamminen K., Hankaniemi M.M., Malm M., Hytönen V.P., Blazevic V. (2022). Antigenicity and immunogenicity of HA2 and M2e influenza virus antigens conjugated to norovirus-like, VP1 capsid-based particles by the SpyTag/SpyCatcher technology. Virology.

[40] Gomes K.B., Allotey-Babington G.L., D’Sa S., Kang S.M., D’Souza M.J. (2022). Dendritic cell activation by a micro particulate based system containing the influenza matrix-2 protein virus-like particle (M2e VLP). Int. J. Pharm..

[41] Braz Gomes K., D’Sa S., Allotey-Babington G.L., Kang S.M., D’Souza M.J. (2021). Transdermal Vaccination with the Matrix-2 Protein Virus-like Particle (M2e VLP) Induces Immunity in Mice against Influenza A Virus. Vaccines.

[42] Gomes K.B., Menon I., Bagwe P., Bajaj L., Kang S.M., D’Souza M.J. (2022). Enhanced Immunogenicity of an Influenza Ectodomain Matrix-2 Protein Virus-like Particle (M2e VLP) Using Polymeric Microparticles for Vaccine Delivery. Viruses.

[43] Kim K.H., Li Z., Bhatnagar N., Subbiah J., Park B.R., Shin C.H., Pushko P., Wang B.Z., Kang S.M. (2022). Universal protection against influenza viruses by multi-subtype neuraminidase and M2 ectodomain virus-like particle. PLoS Pathog..

[44] Hong J.Y., Chen T.H., Chen Y.J., Liu C.C., Jan J.T., Wu S.C. (2019). Highly immunogenic influenza virus-like particles containing B-cell-activating factor (BAFF) for multi-subtype vaccine development. Antiviral. Res..

[45] Maegawa K., Sugita S., Arasaki Y., Nerome R., Nerome K. (2020). Interleukin 12-containing influenza virus-like-particle vaccine elevate its protective activity against heterotypic influenza virus infection. Heliyon.

[46] Makarkov A.I., Golizeh M., Ruiz-Lancheros E., Gopal A.A., Costas-Cancelas I.N., Chierzi S., Pillet S., Charland N., Landry N., Rouiller I. (2019). Plant-derived virus-like particle vaccines drive cross-presentation of influenza A hemagglutinin peptides by human monocyte-derived macrophages. NPJ Vaccines.

[47] Ko E.J., Lee Y., Lee Y.T., Jung Y.J., Ngo V.L., Kim M.C., Kim K.H., Wang B.Z., Gewirtz A.T., Kang S.M. (2019). Flagellin-expressing virus-like particles exhibit adjuvant effects on promoting IgG isotype-switched long-lasting antibody induction and protection of influenza vaccines in CD4-deficient mice. Vaccine.

[48] Zhao Y., Li Z., Voyer J., Li Y., Chen X. (2022). Flagellin/Virus-like Particle Hybrid Platform with High Immunogenicity, Safety, and Versatility for Vaccine Development. ACS Appl. Mater. Interfaces.

[49] Hendin H.E., Lavoie P.O., Gravett J.M., Pillet S., Saxena P., Landry N., D’Aoust M.A., Ward B.J. (2022). Elimination of receptor binding by influenza hemagglutinin improves vaccine-induced immunity. NPJ Vaccines.

[50] Carvalho S.B., Silva R.J.S., Moleirinho M.G., Cunha B., Moreira A.S., Xenopoulos A., Alves P.M., Carrondo M.J.T., Peixoto C. (2019). Membrane-Based Approach for the Downstream Processing of Influenza Virus-Like Particles. Biotechnol. J..

[51] Lai C.C., Cheng Y.C., Chen P.W., Lin T.H., Tzeng T.T., Lu C.C., Lee M.S., Hu A.Y. (2019). Process development for pandemic influenza VLP vaccine production using a baculovirus expression system. J. Biol. Eng..

[52] Zak A.J., Hill B.D., Rizvi S.M., Smith M.R., Yang M., Wen F. (2019). Enhancing the Yield and Quality of Influenza Virus-like Particles (VLPs) Produced in Insect Cells by Inhibiting Cytopathic Effects of Matrix Protein M2. ACS Synth. Biol..

[53] Cohen J. (2013). Immunology. A once-in-a-lifetime flu shot?. Science.

[54] Kanekiyo M., Wei C.J., Yassine H.M., McTamney P.M., Boyington J.C., Whittle J.R., Rao S.S., Kong W.P., Wang L., Nabel G.J. (2013). Self-assembling influenza nanoparticle vaccines elicit broadly neutralizing H1N1 antibodies. Nature.

[55] Yassine H.M., Boyington J.C., McTamney P.M., Wei C.J., Kanekiyo M., Kong W.P., Gallagher J.R., Wang L., Zhang Y., Joyce M.G. (2015). Hemagglutinin-stem nanoparticles generate heterosubtypic influenza protection. Nat. Med..

[56] Darricarrère N., Pougatcheva S., Duan X., Rudicell R.S., Chou T.H., DiNapoli J., Ross T.M., Alefantis T., Vogel T.U., Kleanthous H. (2018). Development of a Pan-H1 Influenza Vaccine. J. Virol..

[57] Darricarrère N., Qiu Y., Kanekiyo M., Creanga A., Gillespie R.A., Moin S.M., Saleh J., Sancho J., Chou T.H., Zhou Y. (2021). Broad neutralization of H1 and H3 viruses by adjuvanted influenza HA stem vaccines in nonhuman primates. Sci. Transl. Med..

[58] Landry N., Ward B.J., Trépanier S., Montomoli E., Dargis M., Lapini G., Vézina L.P. (2010). Preclinical and clinical development of plant-made virus-like particle vaccine against avian H5N1 influenza. PLoS ONE.

[59] Khurana S., Wu J., Verma N., Verma S., Raghunandan R., Manischewitz J., King L.R., Kpamegan E., Pincus S., Smith G. (2011). H5N1 virus-like particle vaccine elicits cross-reactive neutralizing antibodies that preferentially bind to the oligomeric form of influenza virus hemagglutinin in humans. J. Virol..

[60] Fries L.F., Smith G.E., Glenn G.M. (2013). A recombinant viruslike particle influenza A (H7N9) vaccine. N. Engl. J. Med..

[61] Chung K.Y., Coyle E.M., Jani D., King L.R., Bhardwaj R., Fries L., Smith G., Glenn G., Golding H., Khurana S. (2015). ISCOMATRIX™ adjuvant promotes epitope spreading and antibody affinity maturation of influenza A H7N9 virus like particle vaccine that correlate with virus neutralization in humans. Vaccine.

[62] Low J.G., Lee L.S., Ooi E.E., Ethirajulu K., Yeo P., Matter A., Connolly J.E., Skibinski D.A., Saudan P., Bachmann M. (2014). Safety and immunogenicity of a virus-like particle pandemic influenza A (H1N1) 2009 vaccine: Results from a double-blinded, randomized Phase I clinical trial in healthy Asian volunteers. Vaccine.

[63] López-Macías C., Ferat-Osorio E., Tenorio-Calvo A., Isibasi A., Talavera J., Arteaga-Ruiz O., Arriaga-Pizano L., Hickman S.P., Allende M., Lenhard K. (2011). Safety and immunogenicity of a virus-like particle pandemic influenza A (H1N1) 2009 vaccine in a blinded, randomized, placebo-controlled trial of adults in Mexico. Vaccine.

[64] Valero-Pacheco N., Pérez-Toledo M., Villasís-Keever M., Núñez-Valencia A., Boscó-Gárate I., Lozano-Dubernard B., Lara-Puente H., Espitia C., Alpuche-Aranda C., Bonifaz L.C. (2016). Antibody Persistence in Adults Two Years after Vaccination with an H1N1 2009 Pandemic Influenza Virus-Like Particle Vaccine. PLoS ONE.

[65] Pillet S., Aubin É., Trépanier S., Bussière D., Dargis M., Poulin J.F., Yassine-Diab B., Ward B.J., Landry N. (2016). A plant-derived quadrivalent virus like particle influenza vaccine induces cross-reactive antibody and T cell response in healthy adults. Clin. Immunol..

[66] Pillet S., Couillard J., Trépanier S., Poulin J.F., Yassine-Diab B., Guy B., Ward B.J., Landry N. (2019). Immunogenicity and safety of a quadrivalent plant-derived virus like particle influenza vaccine candidate-Two randomized Phase II clinical trials in 18 to 49 and ≥50 years old adults. PLoS ONE.

[67] Ward B.J., Makarkov A., Séguin A., Pillet S., Trépanier S., Dhaliwall J., Libman M.D., Vesikari T., Landry N. (2020). Efficacy, immunogenicity, and safety of a plant-derived, quadrivalent, virus-like particle influenza vaccine in adults (18–64 years) and older adults (≥65 years): Two multicentre, randomised phase 3 trials. Lancet.

[68] Ward B.J., Séguin A., Couillard J., Trépanier S., Landry N. (2021). Phase III: Randomized observer-blind trial to evaluate lot-to-lot consistency of a new plant-derived quadrivalent virus like particle influenza vaccine in adults 18–49 years of age. Vaccine.

[69] Shinde V., Cho I., Plested J.S., Agrawal S., Fiske J., Cai R., Zhou H., Pham X., Zhu M., Cloney-Clark S. (2022). Comparison of the safety and immunogenicity of a novel Matrix-M-adjuvanted nanoparticle influenza vaccine with a quadrivalent seasonal influenza vaccine in older adults: A phase 3 randomised controlled trial. Lancet Infect Dis..

[70] Houser K.V., Chen G.L., Carter C., Crank M.C., Nguyen T.A., Burgos Florez M.C., Berkowitz N.M., Mendoza F., Hendel C.S., Gordon I.J. (2022). Safety and immunogenicity of a ferritin nanoparticle H2 influenza vaccine in healthy adults: A phase 1 trial. Nat. Med..

[71] Battles M.B., McLellan J.S. (2019). Respiratory syncytial virus entry and how to block it. Nat. Rev. Microbiol..

[72] Shi T., McAllister D.A., O’Brien K.L., Simoes E.A.F., Madhi S.A., Gessner B.D., Polack F.P., Balsells E., Acacio S., Aguayo C. (2017). Global, regional, and national disease burden estimates of acute lower respiratory infections due to respiratory syncytial virus in young children in 2015: A systematic review and modelling study. Lancet.

[73] Ivey K.S., Edwards K.M., Talbot H.K. (2018). Respiratory Syncytial Virus and Associations With Cardiovascular Disease in Adults. J. Am. Coll. Cardiol..

[74] Nguyen-Van-Tam J.S., O’Leary M., Martin E.T., Heijnen E., Callendret B., Fleischhackl R., Comeaux C., Tran T.M.P., Weber K. (2022). Burden of respiratory syncytial virus infection in older and high-risk adults: A systematic review and meta-analysis of the evidence from developed countries. Eur. Respir. Rev..

[75] Savic M., Penders Y., Shi T., Branche A., Pirçon J.Y. (2022). Respiratory syncytial virus disease burden in adults aged 60 years and older in high-income countries: A systematic literature review and meta-analysis. Influenza Other Respir. Viruses.

[76] Blount R.E., Morris J.A., Savage R.E. (1956). Recovery of cytopathogenic agent from chimpanzees with coryza. Proc. Soc. Exp. Biol. Med..

[77] Chanock R., Roizman B., Myers R. (1957). Recovery from infants with respiratory illness of a virus related to chimpanzee coryza agent (CCA). I. Isolation, properties and characterization. Am. J. Hyg..

[78] Chin J., Magoffin R.L., Shearer L.A., Schieble J.H., Lennette E.H. (1969). Field evaluation of a respiratory syncytial virus vaccine and a trivalent parainfluenza virus vaccine in a pediatric population. Am. J. Epidemiol..

[79] Harvey T.J., Liu W.J., Wang X.J., Linedale R., Jacobs M., Davidson A., Le T.T., Anraku I., Suhrbier A., Shi P.Y. (2004). Tetracycline-inducible packaging cell line for production of flavivirus replicon particles. J. Virol..

[80] Murawski M.R., McGinnes L.W., Finberg R.W., Kurt-Jones E.A., Massare M.J., Smith G., Heaton P.M., Fraire A.E., Morrison T.G. (2010). Newcastle disease virus-like particles containing respiratory syncytial virus G protein induced protection in BALB/c mice, with no evidence of immunopathology. J. Virol..

[81] McGinnes L.W., Gravel K.A., Finberg R.W., Kurt-Jones E.A., Massare M.J., Smith G., Schmidt M.R., Morrison T.G. (2011). Assembly and immunological properties of Newcastle disease virus-like particles containing the respiratory syncytial virus F and G proteins. J. Virol..

[82] Schmidt M.R., McGinnes L.W., Kenward S.A., Willems K.N., Woodland R.T., Morrison T.G. (2012). Long-term and memory immune responses in mice against Newcastle disease virus-like particles containing respiratory syncytial virus glycoprotein ectodomains. J. Virol..

[83] Schickli J.H., Whitacre D.C., Tang R.S., Kaur J., Lawlor H., Peters C.J., Jones J.E., Peterson D.L., McCarthy M.P., Van Nest G. (2015). Palivizumab epitope-displaying virus-like particles protect rodents from RSV challenge. J. Clin. Investig..

[84] Quan F.S., Kim Y., Lee S., Yi H., Kang S.M., Bozja J., Moore M.L., Compans R.W. (2011). Viruslike particle vaccine induces protection against respiratory syncytial virus infection in mice. J. Infect. Dis..

[85] Lee S., Quan F.S., Kwon Y., Sakamoto K., Kang S.M., Compans R.W., Moore M.L. (2014). Additive protection induced by mixed virus-like particles presenting respiratory syncytial virus fusion or attachment glycoproteins. Antiviral. Res..

[86] Schwarz B., Morabito K.M., Ruckwardt T.J., Patterson D.P., Avera J., Miettinen H.M., Graham B.S., Douglas T. (2016). Viruslike Particles Encapsidating Respiratory Syncytial Virus M and M2 Proteins Induce Robust T Cell Responses. ACS Biomater. Sci. Eng..

[87] Walpita P., Johns L.M., Tandon R., Moore M.L. (2015). Mammalian Cell-Derived Respiratory Syncytial Virus-Like Particles Protect the Lower as well as the Upper Respiratory Tract. PLoS ONE.

[88] Magro M., Mas V., Chappell K., Vázquez M., Cano O., Luque D., Terrón M.C., Melero J.A., Palomo C. (2012). Neutralizing antibodies against the preactive form of respiratory syncytial virus fusion protein offer unique possibilities for clinical intervention. Proc. Natl. Acad. Sci. USA.

[89] Cimica V., Boigard H., Bhatia B., Fallon J.T., Alimova A., Gottlieb P., Galarza J.M. (2016). Novel Respiratory Syncytial Virus-Like Particle Vaccine Composed of the Postfusion and Prefusion Conformations of the F Glycoprotein. Clin. Vaccine Immunol..

[90] Cullen L.M., Schmidt M.R., Morrison T.G. (2017). The importance of RSV F protein conformation in VLPs in stimulation of neutralizing antibody titers in mice previously infected with RSV. Hum. Vaccin. Immunother..

[91] McGinnes Cullen L., Schmidt M.R., Morrison T.G. (2019). Effect of Previous Respiratory Syncytial Virus Infection on Murine Immune Responses to F and G Protein-Containing Virus-Like Particles. J. Virol..

[92] Cullen L.M., Blanco J.C., Morrison T.G. (2015). Cotton rat immune responses to virus-like particles containing the pre-fusion form of respiratory syncytial virus fusion protein. J. Transl. Med..

[93] Kim A.R., Lee D.H., Lee S.H., Rubino I., Choi H.J., Quan F.S. (2018). Protection induced by virus-like particle vaccine containing tandem repeat gene of respiratory syncytial virus G protein. PLoS ONE.

[94] Chu K.B., Lee S.H., Kim M.J., Kim A.R., Moon E.K., Quan F.S. (2022). Virus-like particles coexpressing the PreF and Gt antigens of respiratory syncytial virus confer protection in mice. Nanomedicine.

[95] Lei L., Qin H., Luo J., Tan Y., Yang J., Pan Z. (2021). Construction and immunological evaluation of hepatitis B virus core virus-like particles containing multiple antigenic peptides of respiratory syncytial virus. Virus Res..

[96] Hervé P.L., Dhelft V., Zuniga A., Ghasparian A., Rassek O., Yim K.C., Donne N., Lambert P.H., Benhamou P.H., Sampson H.A. (2021). Epicutaneous immunization using synthetic virus-like particles efficiently boosts protective immunity to respiratory syncytial virus. Vaccine.

[97] Taylor G., Wyld S., Valarcher J.F., Guzman E., Thom M., Widdison S., Buchholz U.J. (2014). Recombinant bovine respiratory syncytial virus with deletion of the SH gene induces increased apoptosis and pro-inflammatory cytokines in vitro, and is attenuated and induces protective immunity in calves. J. Gen. Virol..

[98] Schepens B., Sedeyn K., Vande Ginste L., De Baets S., Schotsaert M., Roose K., Houspie L., Van Ranst M., Gilbert B., van Rooijen N. (2014). Protection and mechanism of action of a novel human respiratory syncytial virus vaccine candidate based on the extracellular domain of small hydrophobic protein. EMBO Mol. Med..

[99] Langley J.M., MacDonald L.D., Weir G.M., MacKinnon-Cameron D., Ye L., McNeil S., Schepens B., Saelens X., Stanford M.M., Halperin S.A. (2018). A Respiratory Syncytial Virus Vaccine Based on the Small Hydrophobic Protein Ectodomain Presented With a Novel Lipid-Based Formulation Is Highly Immunogenic and Safe in Adults: A First-in-Humans Study. J. Infect. Dis..

[100] Blanco J.C.G., Pletneva L.M., McGinnes-Cullen L., Otoa R.O., Patel M.C., Fernando L.R., Boukhvalova M.S., Morrison T.G. (2018). Efficacy of a respiratory syncytial virus vaccine candidate in a maternal immunization model. Nat. Commun..

[101] Blanco J.C.G., Cullen L.M., Kamali A., Sylla F.Y.D., Boukhvalova M.S., Morrison T.G. (2021). Evolution of protection after maternal immunization for respiratory syncytial virus in cotton rats. PLoS Pathog..

[102] Cullen L.M., Schmidt M.R., Torres G.M., Capoferri A.A., Morrison T.G. (2019). Comparison of Immune Responses to Different Versions of VLP Associated Stabilized RSV Pre-Fusion F Protein. Vaccines.

[103] Blanco J.C.G., Fernando L.R., Zhang W., Kamali A., Boukhvalova M.S., McGinnes-Cullen L., Morrison T.G. (2019). Alternative Virus-Like Particle-Associated Prefusion F Proteins as Maternal Vaccines for Respiratory Syncytial Virus. J. Virol..

[104] Kwon Y.M., Hwang H.S., Lee Y.T., Kim K.H., Lee Y., Kim M.C., Lee Y.N., Quan F.S., Moore M.L., Kang S.M. (2019). Respiratory Syncytial Virus Fusion Protein-encoding DNA Vaccine Is Less Effective in Conferring Protection against Inflammatory Disease than a Virus-like Particle Platform. Immune Netw..

[105] Kwon Y.M., Lee Y., Kim K.H., Jung Y.J., Li Z., Jeeva S., Lee S., Moore M.L., Kang S.M. (2019). Antigenicity and immunogenicity of unique prefusion-mimic F proteins presented on enveloped virus-like particles. Vaccine.

[106] Huertas-Díaz M.C., Phan S., Elson A., Nuñez I., Wei H., Sakamoto K., He B. (2019). Parainfluenza virus 5 (PIV5) amplifying virus-like particles expressing respiratory syncytial virus (RSV) antigens protect mice against RSV infection. Vaccine.

[107] Swanson K.A., Rainho-Tomko J.N., Williams Z.P., Lanza L., Peredelchuk M., Kishko M., Pavot V., Alamares-Sapuay J., Adhikarla H., Gupta S. (2020). A respiratory syncytial virus (RSV) F protein nanoparticle vaccine focuses antibody responses to a conserved neutralization domain. Sci. Immunol..

[108] Acosta P.L., Caballero M.T., Polack F.P. (2015). Brief History and Characterization of Enhanced Respiratory Syncytial Virus Disease. Clin. Vaccine Immunol..

[109] Lee Y., Lee Y.T., Ko E.J., Kim K.H., Hwang H.S., Park S., Kwon Y.M., Kang S.M. (2017). Soluble F proteins exacerbate pulmonary histopathology after vaccination upon respiratory syncytial virus challenge but not when presented on virus-like particles. Hum. Vaccines Immunother..

[110] Chirkova T., Ha B., Rimawi B.H., Oomens A.G.P., Hartert T.V., Anderson L.J. (2020). In vitro model for the assessment of human immune responses to subunit RSV vaccines. PLoS ONE.

[111] Turi K.N., Shankar J., Anderson L.J., Rajan D., Gaston K., Gebretsadik T., Das S.R., Stone C., Larkin E.K., Rosas-Salazar C. (2018). Infant Viral Respiratory Infection Nasal Immune-Response Patterns and Their Association with Subsequent Childhood Recurrent Wheeze. Am. J. Respir. Crit. Care Med..

[112] Eichinger K.M., Kosanovich J.L., Gidwani S.V., Zomback A., Lipp M.A., Perkins T.N., Oury T.D., Petrovsky N., Marshall C.P., Yondola M.A. (2020). Prefusion RSV F Immunization Elicits Th2-Mediated Lung Pathology in Mice When Formulated With a Th2 (but Not a Th1/Th2-Balanced) Adjuvant Despite Complete Viral Protection. Front. Immunol..

[113] Adams M.J., Lefkowitz E.J., King A.M., Harrach B., Harrison R.L., Knowles N.J., Kropinski A.M., Krupovic M., Kuhn J.H., Mushegian A.R. (2016). Ratification vote on taxonomic proposals to the International Committee on Taxonomy of Viruses (2016). Arch. Virol..

[114] Masters P.S. (2006). The molecular biology of coronaviruses. Adv. Virus Res..

[115] EA J.A., Jones I.M. (2019). Membrane binding proteins of coronaviruses. Future Virol..

[116] Drosten C., Günther S., Preiser W., van der Werf S., Brodt H.R., Becker S., Rabenau H., Panning M., Kolesnikova L., Fouchier R.A. (2003). Identification of a novel coronavirus in patients with severe acute respiratory syndrome. N. Engl. J. Med..

[117] Zaki A.M., van Boheemen S., Bestebroer T.M., Osterhaus A.D., Fouchier R.A. (2012). Isolation of a novel coronavirus from a man with pneumonia in Saudi Arabia. N. Engl. J. Med..

[118] WHO WHO Coronavirus (COVID-19) Dashboard. https://covid19.who.int/.

[119] Xia S., Zhu Y., Liu M., Lan Q., Xu W., Wu Y., Ying T., Liu S., Shi Z., Jiang S. (2020). Fusion mechanism of 2019-nCoV and fusion inhibitors targeting HR1 domain in spike protein. Cell Mol. Immunol..

[120] Geng Q., Tai W., Baxter V.K., Shi J., Wan Y., Zhang X., Montgomery S.A., Taft-Benz S.A., Anderson E.J., Knight A.C. (2021). Novel virus-like nanoparticle vaccine effectively protects animal model from SARS-CoV-2 infection. PLoS Pathog..

[121] Xu R., Shi M., Li J., Song P., Li N. (2020). Construction of SARS-CoV-2 Virus-Like Particles by Mammalian Expression System. Front. Bioeng. Biotechnol..

[122] Tan T.K., Rijal P., Rahikainen R., Keeble A.H., Schimanski L., Hussain S., Harvey R., Hayes J.W.P., Edwards J.C., McLean R.K. (2021). A COVID-19 vaccine candidate using SpyCatcher multimerization of the SARS-CoV-2 spike protein receptor-binding domain induces potent neutralising antibody responses. Nat. Commun..

[123] Sullivan E., Sung P.Y., Wu W., Berry N., Kempster S., Ferguson D., Almond N., Jones I.M., Roy P. (2022). SARS-CoV-2 Virus-like Particles Produced by a Single Recombinant Baculovirus Generate Anti-S Antibody and Protect against Variant Challenge. Viruses.

[124] Chevillard C., Amen A., Besson S., Hannani D., Bally I., Dettling V., Gout E., Moreau C.J., Buisson M., Gallet S. (2022). Elicitation of potent SARS-CoV-2 neutralizing antibody responses through immunization with a versatile adenovirus-inspired multimerization platform. Mol. Ther..

[125] Yang Y., Shi W., Abiona O.M., Nazzari A., Olia A.S., Ou L., Phung E., Stephens T., Tsybovsky Y., Verardi R. (2021). Newcastle Disease Virus-Like Particles Displaying Prefusion-Stabilized SARS-CoV-2 Spikes Elicit Potent Neutralizing Responses. Vaccines.

[126] Liu X., Chang X., Rothen D., Derveni M., Krenger P., Roongta S., Wright E., Vogel M., Tars K., Mohsen M.O. (2021). AP205 VLPs Based on Dimerized Capsid Proteins Accommodate RBM Domain of SARS-CoV-2 and Serve as an Attractive Vaccine Candidate. Vaccines.

[127] Chu K.B., Kang H.J., Yoon K.W., Lee H.A., Moon E.K., Han B.K., Quan F.S. (2021). Influenza Virus-like Particle (VLP) Vaccines Expressing the SARS-CoV-2 S Glycoprotein, S1, or S2 Domains. Vaccines.

[128] Kaewborisuth C., Wanitchang A., Koonpaew S., Srisutthisamphan K., Saenboonrueng J., Im-Erbsin R., Inthawong M., Sunyakumthorn P., Thaweerattanasinp T., Tanwattana N. (2022). Chimeric Virus-like Particle-Based COVID-19 Vaccine Confers Strong Protection against SARS-CoV-2 Viremia in K18-hACE2 Mice. Vaccines.

[129] Chen J., Xu W., Li L., Yi L., Jiang Y., Hao P., Xu Z., Zou W., Li P., Gao Z. (2022). Immunogenicity and protective potential of chimeric virus-like particles containing SARS-CoV-2 spike and H5N1 matrix 1 proteins. Front. Cell Infect Microbiol..

[130] Pillet S., Arunachalam P.S., Andreani G., Golden N., Fontenot J., Aye P.P., Röltgen K., Lehmicke G., Gobeil P., Dubé C. (2022). Safety, immunogenicity, and protection provided by unadjuvanted and adjuvanted formulations of a recombinant plant-derived virus-like particle vaccine candidate for COVID-19 in nonhuman primates. Cell Mol. Immunol..

[131] Volkmann A., Koopman G., Mooij P., Verschoor E.J., Verstrepen B.E., Bogers W., Idorn M., Paludan S.R., Vang S., Nielsen M.A. (2022). A Capsid Virus-Like Particle-Based SARS-CoV-2 Vaccine Induces High Levels of Antibodies and Protects Rhesus Macaques. Front. Immunol..

[132] WHO Tracking SARS-CoV-2 Variants. https://www.who.int/en/activities/tracking-SARS-CoV-2-variants/.

[133] van Oosten L., Altenburg J.J., Fougeroux C., Geertsema C., van den End F., Evers W.A.C., Westphal A.H., Lindhoud S., van den Berg W., Swarts D.C. (2021). Two-Component Nanoparticle Vaccine Displaying Glycosylated Spike S1 Domain Induces Neutralizing Antibody Response against SARS-CoV-2 Variants. mBio.

[134] Fougeroux C., Goksøyr L., Idorn M., Soroka V., Myeni S.K., Dagil R., Janitzek C.M., Søgaard M., Aves K.L., Horsted E.W. (2021). Capsid-like particles decorated with the SARS-CoV-2 receptor-binding domain elicit strong virus neutralization activity. Nat. Commun..

[135] Mohsen M.O., Balke I., Zinkhan S., Zeltina V., Liu X., Chang X., Krenger P.S., Plattner K., Gharailoo Z., Vogt A.S. (2022). A scalable and highly immunogenic virus-like particle-based vaccine against SARS-CoV-2. Allergy.

[136] Chang X., Zeltins A., Mohsen M.O., Gharailoo Z., Zha L., Liu X., Walton S., Vogel M., Bachmann M.F. (2021). A Novel Double Mosaic Virus-like Particle-Based Vaccine against SARS-CoV-2 Incorporates Both Receptor Binding Motif (RBM) and Fusion Domain. Vaccines.

[137] Chang X., Liu X., Mohsen M.O., Zeltins A., Martina B., Vogel M., Bachmann M.F. (2022). Induction of Broadly Cross-Reactive Antibodies by Displaying Receptor Binding Domains of SARS-CoV-2 on Virus-like Particles. Vaccines.

[138] Syed A.M., Ciling A., Taha T.Y., Chen I.P., Khalid M.M., Sreekumar B., Chen P.Y., Kumar G.R., Suryawanshi R., Silva I. (2022). Omicron mutations enhance infectivity and reduce antibody neutralization of SARS-CoV-2 virus-like particles. Proc. Natl. Acad. Sci. USA.

[139] Servellita V., Syed A.M., Morris M.K., Brazer N., Saldhi P., Garcia-Knight M., Sreekumar B., Khalid M.M., Ciling A., Chen P.Y. (2022). Neutralizing immunity in vaccine breakthrough infections from the SARS-CoV-2 Omicron and Delta variants. Cell.

[140] Chan S.K., Du P., Ignacio C., Mehta S., Newton I.G., Steinmetz N.F. (2021). Biomimetic Virus-Like Particles as Severe Acute Respiratory Syndrome Coronavirus 2 Diagnostic Tools. ACS Nano.

[141] Bommireddy R., Stone S., Bhatnagar N., Kumari P., Munoz L.E., Oh J., Kim K.H., Berry J.T.L., Jacobsen K.M., Jaafar L. (2022). Influenza Virus-like Particle-Based Hybrid Vaccine Containing RBD Induces Immunity against Influenza and SARS-CoV-2 Viruses. Vaccines.

[142] Chauveau L., Bridgeman A., Tan T.K., Beveridge R., Frost J.N., Rijal P., Pedroza-Pacheco I., Partridge T., Gilbert-Jaramillo J., Knight M.L. (2021). Inclusion of cGAMP within virus-like particle vaccines enhances their immunogenicity. EMBO Rep..

[143] Dalvie N.C., Rodriguez-Aponte S.A., Hartwell B.L., Tostanoski L.H., Biedermann A.M., Crowell L.E., Kaur K., Kumru O.S., Carter L., Yu J. (2021). Engineered SARS-CoV-2 receptor binding domain improves manufacturability in yeast and immunogenicity in mice. Proc. Natl. Acad. Sci. USA.

[144] Wong T.Y., Russ B.P., Lee K.S., Miller O.A., Kang J., Cooper M., Winters M.T., Rodriguez-Aponte S.A., Dalvie N.C., Johnston R.S. (2022). RBD-VLP Vaccines Adjuvanted with Alum or SWE Protect K18-hACE2 Mice against SARS-CoV-2 VOC Challenge. mSphere.

[145] Vogt A.S., Jörg L., Martina B., Krenger P.S., Chang X., Zeltins A., Vogel M., Mohsen M.O., Bachmann M.F. (2022). Virus-Like Particles Are Efficient Tools for Boosting mRNA-Induced Antibodies. Front. Immunol..

[146] Powell A.E., Zhang K., Sanyal M., Tang S., Weidenbacher P.A., Li S., Pham T.D., Pak J.E., Chiu W., Kim P.S. (2021). A Single Immunization with Spike-Functionalized Ferritin Vaccines Elicits Neutralizing Antibody Responses against SARS-CoV-2 in Mice. ACS Cent. Sci..

[147] Joyce M.G., Chen W.H., Sankhala R.S., Hajduczki A., Thomas P.V., Choe M., Martinez E.J., Chang W.C., Peterson C.E., Morrison E.B. (2021). SARS-CoV-2 ferritin nanoparticle vaccines elicit broad SARS coronavirus immunogenicity. Cell Rep..

[148] Mohsen M.O., Bachmann M.F. (2022). Virus-like particle vaccinology, from bench to bedside. Cell Mol. Immunol..

[149] Ward B.J., Gobeil P., Séguin A., Atkins J., Boulay I., Charbonneau P.Y., Couture M., D’Aoust M.A., Dhaliwall J., Finkle C. (2021). Phase 1 randomized trial of a plant-derived virus-like particle vaccine for COVID-19. Nat. Med..

[150] Hager K.J., Pérez Marc G., Gobeil P., Diaz R.S., Heizer G., Llapur C., Makarkov A.I., Vasconcellos E., Pillet S., Riera F. (2022). Efficacy and Safety of a Recombinant Plant-Based Adjuvanted Covid-19 Vaccine. N. Engl. J. Med..

[151] Le Tallec D., Doucet D., Elouahabi A., Harvengt P., Deschuyteneer M., Deschamps M. (2009). Cervarix, the GSK HPV-16/HPV-18 AS04-adjuvanted cervical cancer vaccine, demonstrates stability upon long-term storage and under simulated cold chain break conditions. Hum. Vaccin..

[152] Correia R., Meneses L., Richheimer C., Alves P.M., Carrondo M.J.T., Duarte A.R.C., Paiva A., Roldão A. (2021). Improved storage of influenza HA-VLPs using a trehalose-glycerol natural deep eutectic solvent system. Vaccine.

[153] Aves K.L., Janitzek C.M., Fougeroux C.E., Theander T.G., Sander A.F. (2022). Freeze-Drying of a Capsid Virus-like Particle-Based Platform Allows Stable Storage of Vaccines at Ambient Temperature. Pharmaceutics.

[154] Serradell M.C., Rupil L.L., Martino R.A., Prucca C.G., Carranza P.G., Saura A., Fernández E.A., Gargantini P.R., Tenaglia A.H., Petiti J.P. (2019). Efficient oral vaccination by bioengineering virus-like particles with protozoan surface proteins. Nat. Commun..

[155] Bellier B., Saura A., Luján L.A., Molina C.R., Luján H.D., Klatzmann D. (2022). A Thermostable Oral SARS-CoV-2 Vaccine Induces Mucosal and Protective Immunity. Front. Immunol..

